# Specific Silencing of Microglial Gene Expression in the Rat Brain by Nanoparticle-Based Small Interfering RNA Delivery

**DOI:** 10.1021/acsami.1c22434

**Published:** 2022-01-18

**Authors:** Shanshan Guo, Fernando Cázarez-Márquez, Han Jiao, Ewout Foppen, Nikita L. Korpel, Anita E. Grootemaat, Nalan Liv, Yuanqing Gao, Nicole van der Wel, Bing Zhou, Guangjun Nie, Chun-Xia Yi

**Affiliations:** † Department of Endocrinology and Metabolism, Laboratory of Endocrinology, Amsterdam Gastroenterology Endocrinology Metabolism Research Institute, Amsterdam University Medical Centre (UMC), location AMC, University of Amsterdam, 1105 AZ Amsterdam, The Netherlands; ‡ Key Laboratory of Cardiovascular and Cerebrovascular Medicine, School of Pharmacy, Nanjing Medical University, Nanjing 211166, China; § Netherlands Institute for Neuroscience, Institute of the Royal Netherlands Academy of Arts and Sciences, 1105 AZ Amsterdam, The Netherlands; ∥ Cellular Imaging Core Facility, Amsterdam University Medical Centre (UMC), location AMC, University of Amsterdam, 1105 AZ Amsterdam, The Netherlands; ⊥ Section Cell Biology, Center for Molecular Medicine, University Medical Center Utrecht, 3584 CX Utrecht, The Netherlands; # Institute of Synthetic Biology, Shenzhen Institutes of Advanced Technology, Chinese Academy of Sciences, Shenzhen 518055, China; ∇ CAS Key Laboratory for Biomedical Effects of Nanomaterials and Nanosafety, National Center for Nanoscience and Technology, Beijing 100190, China

**Keywords:** nanoparticles, microglia, hypothalamus, phagocytosis, siRNA, CD11b, TLR4

## Abstract

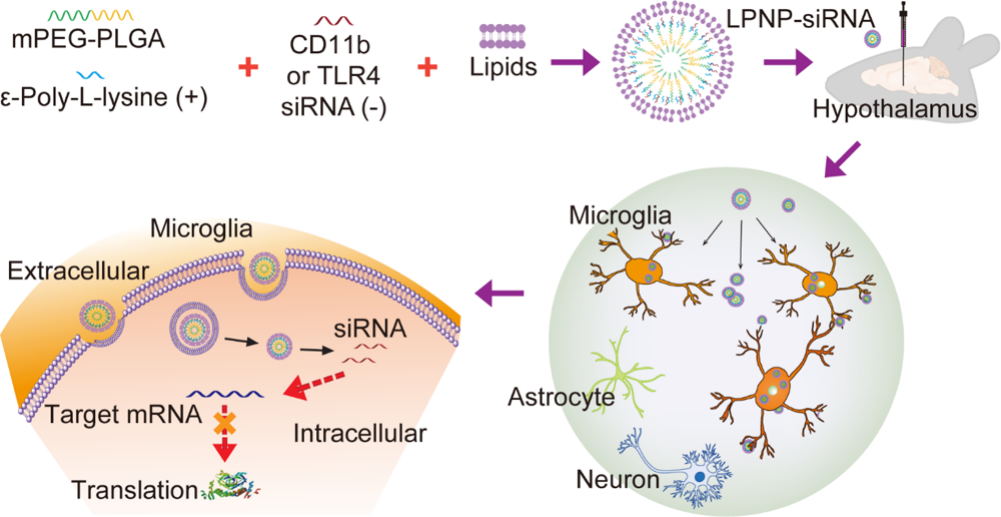

Microglia are the major innate immune cells in the brain and are essential for maintaining homeostasis in a neuronal microenvironment. Currently, a genetic tool to modify microglial gene expression in specific brain regions is not available. In this report, we introduce a tailor-designed method that uses lipid and polymer hybridized nanoparticles (LPNPs) for the local delivery of small interfering RNAs (siRNAs), allowing the silencing of specific microglial genes in the hypothalamus. Our physical characterization proved that this LPNP-siRNA was uniform and stable. We demonstrated that, due to their natural phagocytic behavior, microglial cells are the dominant cell type taking up these LPNPs in the hypothalamus of rats. We then tested the silencing efficiency of LPNPs carrying a cluster of differentiation molecule 11b (CD11b) or Toll-like receptor 4 (TLR4) siRNA using different *in vivo* and *in vitro* approaches. In cultured microglial cells treated with LPNP-CD11b siRNA or LPNP-TLR4 siRNA, we found a silencing efficiency at protein expression levels of 65 or 77%, respectively. In line with this finding, immunohistochemistry and western blotting results from *in vivo* experiments showed that LPNP-CD11b siRNA significantly inhibited microglial CD11b protein expression in the hypothalamus. Furthermore, following lipopolysaccharide (LPS) stimulation of cultured microglial cells, gene expression of the TLR4 downstream signaling component myeloid differentiation factor 88 and its associated cytokines was significantly inhibited in LPNP-TLR4 siRNA-treated microglial cells compared with cells treated with LPNP-scrambled siRNA. Finally, after LPNP-TLR4 siRNA injection into the rat hypothalamus, we observed a significant reduction in microglial activation in response to LPS compared with the control rats injected with LPNP-scrambled siRNA. Our results indicate that LPNP-siRNA is a promising tool to manipulate microglial activity locally in the brain and may serve as a prophylactic approach to prevent microglial dysfunction-associated diseases.

## Introduction

Microglia are long-surviving and self-renewing innate immune cells in the central nervous system. The major functions of microglia are clearing debris and invading pathogens to maintain a healthy microenvironment that enables adequate neuronal activity.^1−5^ Recent genome-wide analyses have revealed that microglia show a distinctive and brain region-dependent transcriptional identity with clear differences in bioenergetic and immune-regulatory pathways.^6^ Current approaches available for genetic manipulation of microglia largely depend on the inducible Cre–loxP recombination system, in which the Cre recombinase gene is mostly driven by the monocyte promoter Cx3cr1. However, this approach modifies microglial gene expression in the entire central nervous system.^7−10^ Also, other approaches, such as pharmacological tools that are effective in modifying microglial proliferation and activity, using inhibitors of colony-stimulating factor-1 (CSF1R) or minocycline,^11−13^ are usually administered systemically without any brain region specificity. Viral approaches that have proven to be effective in manipulating genes in neurons and astrocytes have substantial difficulties with transducing microglial cells, likely due to the natural immune protection behavior of microglial cells.^14^ Therefore, thus far, an approach enabling brain region-specific modulation of microglial activity is not available.

Microglia are natural phagocytes; therefore, we investigated whether their phagocytic behavior would allow the engulfment of biocompatible and biodegradable nanomaterials and, if so, whether this approach would allow the local delivery of small interfering RNAs (siRNAs) to selectively inhibit target gene expression in microglial cells in a specific brain area.^15^ To this end, we absorbed the siRNAs to the surface of the cationic ε-polylysine copolymer nanoparticles (NPs) and coated the NPs with a lipid film. This lipid and polymer hybridized nanocarrier (LPNP), which can deliver siRNAs (LPNP-siRNA), allowed us to specifically and locally silence two genes commonly expressed by microglia, CD11b (also known as integrin subunit alpha M, ITGAM) and Toll-like receptor 4 (TLR4).

In the central nervous system, the hypothalamus is primarily responsible for the regulation of whole-body energy homeostasis. Malfunction of the hypothalamic network has been identified as a major mechanism for metabolic syndrome development.^16−19^ Microglial cells in this brain region respond rapidly and robustly to metabolic stress or other endocrine function-associated immune challenges, including the saturated fatty acid-activated TLR4 signaling pathway.^8,9,20−24^ Therefore, to establish our novel method and study this important microglial population in the brain, we targeted our LPNP-siRNA to microglial cells in the hypothalamus.

First, by encapsulating fluorophore-labeled nanogold particles inside the LPNPs, we demonstrated that the microglia are the dominant cell type in the rat brain that take up LPNPs. Next, we tested the gene silencing efficiency of LPNP-siRNA *in vitro* and *in vivo*. In cultured microglial cells, both LPNP-CD11b siRNA and LPNP-TLR4 siRNA efficiently silenced CD11b and TLR4 protein expression. The silencing efficiency of LPNP-CD11b siRNA was also confirmed *in vivo*. Finally, we tested the effectiveness of LPNP-TLR4 siRNA by infusing it into the hypothalamus and challenging the animal with the TLR4 ligand lipopolysaccharide (LPS) to mimic an endogenous immune stimulation. Microglial reactivity was reduced in LPNP-TLR4 siRNA-treated animals after LPS stimulation. Together, these results suggest that LPNP-siRNA is a promising tool for the spatial manipulation of microglial activity in the brain, and it may serve as a prophylactic approach to prevent microglial dysfunction-associated diseases.

## Results and Discussion

### Characterization of LPNP-siRNA by Assessing Morphology and Cytotoxicity

**Figure 1:**
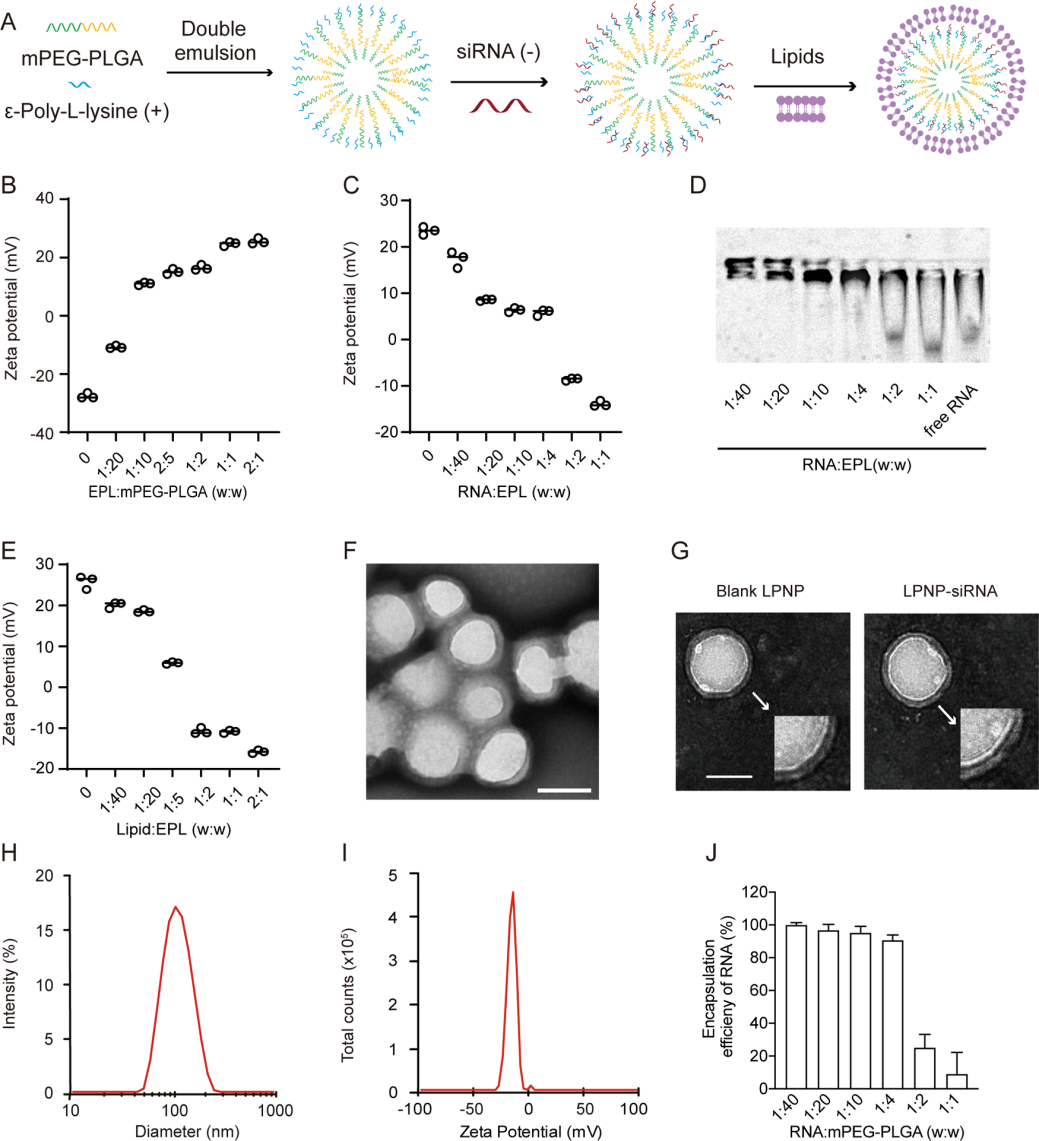
Characterization of LPNP-siRNA. (A) Preparation of LPNP-siRNA. (B) Zeta potential changes of nanoparticles after modification with different amounts of ε-poly-l-lysine (EPL). (C) Zeta potential changes of EPL-modified nanoparticles loaded with different amounts of RNA. (D) Electrophoretic mobility of RNA absorbed by EPL-modified nanoparticles. (E) Changes in the zeta potentials of LPNPs after coating with a lipid bilayer shell at different mass ratios (EPL/lipid). (F, G) Transmission electron microscopy (TEM) images of LPNP-siRNA particles and blank LPNPs; the spherical core–shell structures of the blank LPNP and LPNP-siRNA particles are shown in panel (F). (H, I) Diameter and zeta potential of LPNP-siRNA as determined by dynamic light scattering. (J) Encapsulation efficiency of RNA at various RNA/mPEG-PLGA weight ratio (*n* = 3). All the results are presented as means ± SD. Scale bar: 100 nm in panels (F, G).

The designed nanoparticle-based siRNAs comprised a cationic copolymer nanocore (NPs) and a lipid bilayer shell (Figure 1A). The NPs were generated using the double-emulsion method with the mPEG-PLGA monomers and ε-poly-l-lysine (EPL) (Figure 1A). The negatively charged CD11b siRNA, TLR4 siRNA, or scrambled control siRNA was absorbed onto the surface of the NPs. The lipid bilayer shell was designed to protect the siRNA from environmental nucleases before being taken up by microglia *in vivo*. Moreover, the favorable character of the lipid shell prevented the aggregation of nanoparticles and accelerated phagocytosis into the cells.^25,26^

We first examined the surface charge density of EPL-modified mPEG-PLGA nanoparticles with different weight ratios of EPL:mPEG-PLGA to assess the EPL binding ability at the copolymer nanoparticle surface. The results showed that EPL effectively bound at the surface of the nanoparticles as the zeta potential of the EPL-modified nanoparticles changed radically, from −27.6 to 25.6 mV, when EPL was increased in mass ratio (Figure 1B). The RNA-binding capacity of EPL-modified nanoparticles was evaluated by using these nanoparticles to absorb RNA. When RNA was mixed with EPL-modified nanoparticles in different proportions, the zeta potential of EPL-modified nanoparticles changed from 23.47 to −13.9 mV along the increased mass ratio of RNA:EPL (Figure 1C). These results could be observed in an electromobility shift assay more intuitionally (Figure 1D). RNA was neutralized in agarose gel after being mixed with ENPs at various RNA:EPL ratios. These results demonstrated that the EPL-modified nanoparticles in our study had a large capacity to absorb negatively charged nucleic acids. Next, we examined whether a PEGylated lipid bilayer film could assemble effectively on the surface of EPL-modified nanoparticles. We observed when the weight ratio of lipid:EPL was increased from 0 to 2:1; the zeta potential of EPL-modified nanoparticles was gradually decreased from 25.8 to −15.8 mV (Figure 1E).

The morphology and structure of the siRNA-encapsulated LPNPs were also characterized using transmission electron microscopy (TEM). All the hybrid nanoparticles were dispersed, with a well-defined spherical core–shell structure (Figure 1F,G). The average size of the LPNP-siRNA was 102.1 nm, with the 0.2 polydispersity index (PDI) value (Figure 1H), and the average surface charge of the LPNP-siRNA was −17.2 mV (Figure 1I). A previous study has shown that these types of LPNPs are highly stable in serum.^27^ Both the mobilization of the bands (Figure 1D) and the determination of encapsulation efficiency (Figure 1J) showed that the RNA was almost fully absorbed onto the NPs when the RNA and polymer weight ratio was between 1:40 and 1:4.

The materials used to make the LPNPs (mPEG-PLGA, phospholipids, and cholesterol) have been demonstrated to be biologically safe in previous studies.^28−30^ We also evaluated the safety of the LPNPs in our preparation before applying them in *in vitro* and *in vivo* experiments. First, we examined whether LPNPs cause toxicity or an immune response in microglial cells in culture. Our results showed that the viability of BV2 cells (a microglial cell line) treated for 24 h with LPNPs at different concentrations did not change compared with that of the control group (non-treated BV2 cells) (Figure S1A). Moreover, LPNPs did not change the gene expression of microglial cytokines such as TNFα, IL-6, and IL-1β (Figure S1B–D).

### Microglial Cells Can Take Up, Disassemble, and Degrade LPNPs

Next, we performed time-course studies to characterize the fate of LPNPs after their uptake by microglial cells. We encapsulated the fluorescent dye rhodamine B in LPNPs (LPNP-RhoB) and traced the RhoB fluorescence signals in microglial cells in culture. After treatment with LPNP-RhoB for 0.5, 1, 2, 4, and 24 h, the relative RhoB fluorescence intensity per cell increased continuously (Figure S2), indicating that these microglial cells could phagocytize LPNPs incessantly within 24 h. In a separate study, we replaced the nanoparticle-containing media with fresh media after treating the microglial cells with LPNP-RhoB for 0.5, 1, 2, and 4 h. Sixteen hours later, most of the fluorescence signals had disappeared (Figure S3), indicating the release and elimination of rhodamine B from the LPNPs by the cultured microglial cells.

### Microglia, but Not Neurons or Astrocytes, Take Up LPNPs in Rat Brains

To demonstrate the specific uptake of the LPNPs by the microglial cells *in vivo*, we injected the LPNP-RhoB into the lateral hypothalamus in the rat brain. Rats were sacrificed by perfusion fixation 4 h after the injection. By immunofluorescence staining, we found specific accumulation of LPNP-RhoB in microglia, whereas no LPNP-RhoB was found in astrocytes or orexin-expressing neurons (Figure S4). In addition, we found no significant differences in microglial morphology (as indicated by ionized calcium-binding adaptor molecule 1 immunoreactivity (Iba1-ir)) in the brain region that received the vehicle or LPNP injection (Figure S5).

**Figure 2:**
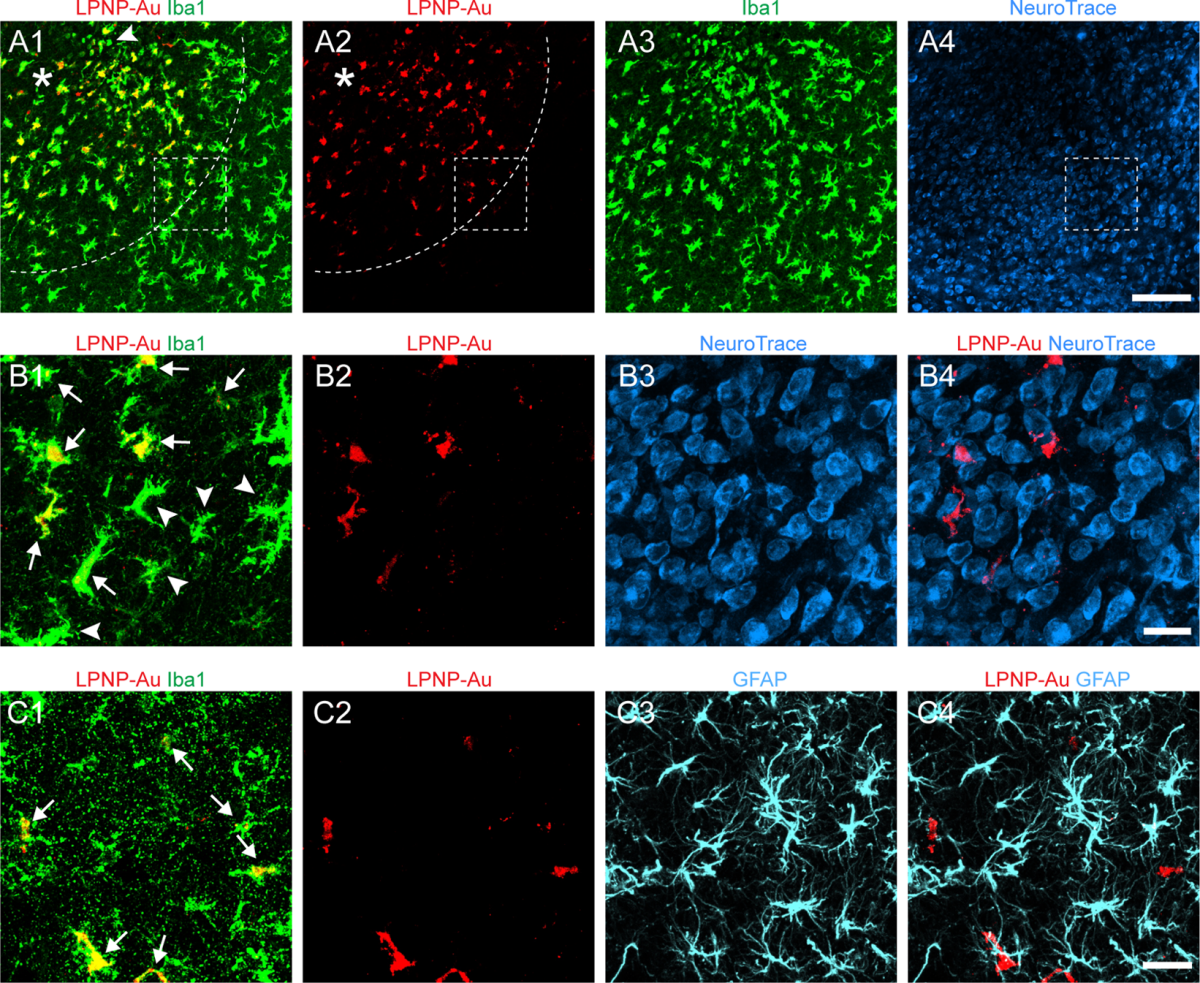
Specific accumulation of LPNP-Au-555 in microglial cells in the rat hypothalamus. (A1–A3) Colocalization of LPNP-Au-555 (red) and ionized calcium-binding adaptor molecule 1 immunoreactivity (Iba1-ir, green) in microglial cells. The dotted lines in panels (A1, A2) show the diffusion of LPNP-Au-555 at a radius of ≈300 μm around the injection spot (asterisk in panels A1, A2). An arrowhead in panel (A1) shows an Iba1-ir microglial cell that does not contain LPNP-Au. (A4) NeuroTrace histochemical staining (blue) in the same area of panels (A1–A3). (B1–B4) High-magnification images of the area framed in panels (A1, A2, A4). Arrows in panel (B1) show Iba1-ir microglia containing LPNP-Au-555. Microglia located at the edge of the area diffused by LPNP-Au-555 are indicated by arrowheads in panel (B1). Panels (B3, B4) show no accumulation of LPNP-Au-555 in the NeuroTrace-stained neurons. (C1–C4) No accumulation of LPNP-Au-555 is observed in glial fibrillary acidic protein-ir (GFAP-ir) astrocytes (cyan). Arrows in panel (C1) show microglia containing both LPNP-Au-555 and Iba1-ir. *n* = 4. Scale bar: 100 μm in panels (A1–A4) and 20 μm in panels (B1–B4) and (C1–C4).

To further demonstrate the specific uptake of LPNP by microglia *in vivo* and prevent difficulties in quantifying the absolute amount of LPNP-RhoB accumulated in microglia due to the constant diminishing RhoB fluorescence, we used fluorescence-conjugated gold particles instead of RhoB to label the LPNPs. We expect that phagolysosomes will not be able to digest gold nanoparticles because of the stability of gold,^31,32^ allowing us to characterize the phagocytic capacity by measuring the total amount of gold nanoparticles accumulated in microglial cells following LPNP treatment. We encapsulated Alexa-555-conjugated 10 nm gold nanoparticles (Au-555) into LPNPs (LPNP-Au-555). These gold nanoparticle-containing LPNPs had a similar size and surface charge as the LPNP-siRNA (Figure S6). We injected these LPNP-Au-555 into the hypothalamus of rats, and the rats were sacrificed by perfusion fixation 24 h after the injection. Confocal microscopy revealed that LPNP-Au-555 diffused around the injection spot (Figure 2A1) at a radius of ≈300 μm (Figure 2A2). We found the LPNP-Au-555 was specifically accumulated in Iba1-ir microglial cells (Figure 2A1−A3,B1,B2), except few microglial cells (6.1 ± 0.8%) among the others did not contain LPNP-Au-555 (Figure 2A1). Moreover, no LPNP-Au-555 fluorescence signals were detected in neurons (as indicated by NeuroTrace staining) (Figure 2A4,B3,B4) or in astrocytes (Figure 2C1–C4). Thus, we concluded that microglia are the dominant cells in the brain that take up the LPNP.

**Figure 3:**
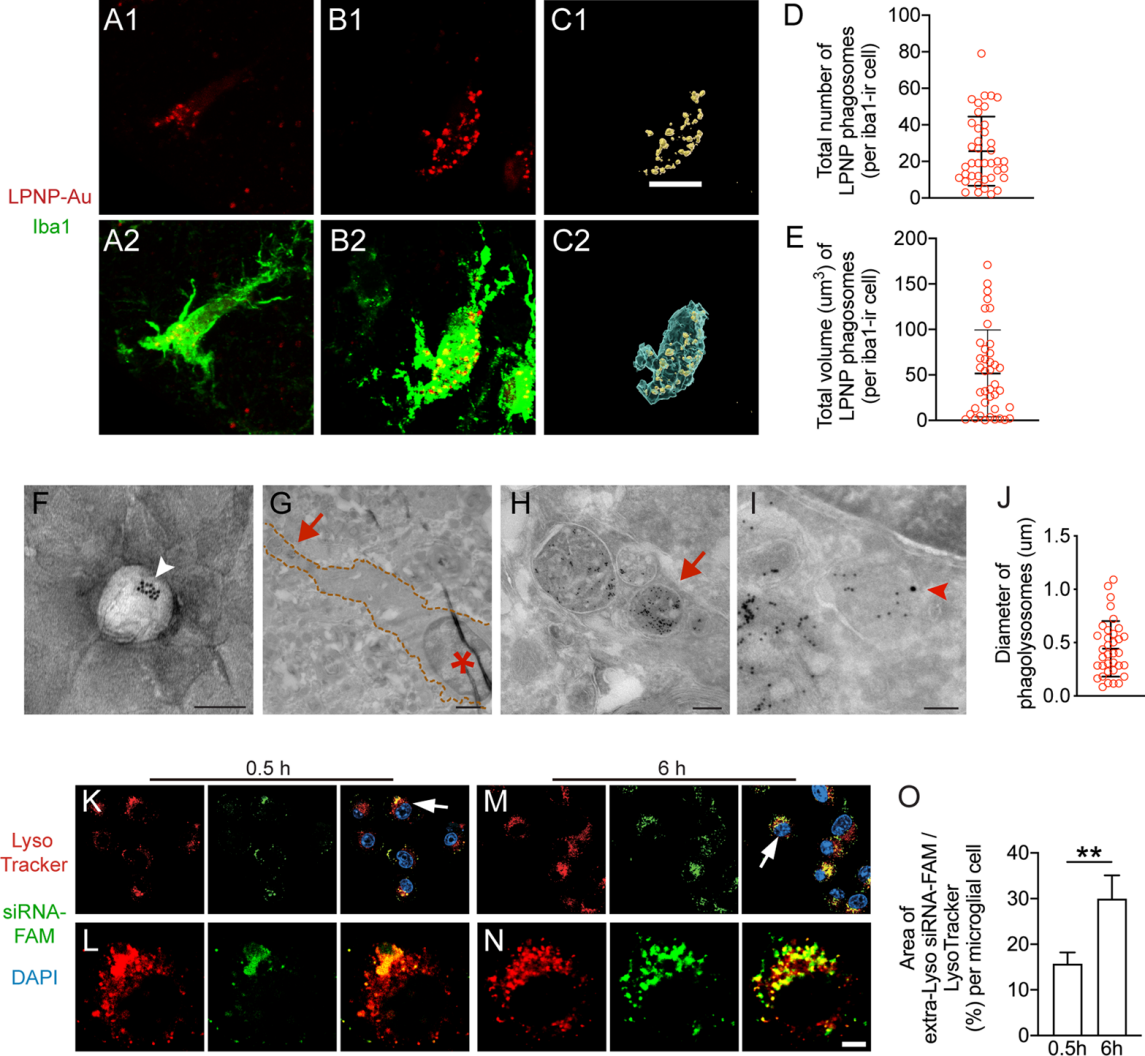
Characterization of the microglial capacity to phagocytize LPNPs in the rat hypothalamus. (A, B) Small (A) or large (B) number of LPNP-Au-containing phagolysosomes (red) accumulate in ionized calcium binding adaptor molecule 1-immunoreactive (Iba1-ir, green) microglia. (C) 3D reconstruction of LPNP-Au-555-containing phagolysosomes (yellow) and Iba1-ir microglial soma (blue) based on panels (B1, B2) for the volume calculation. (D, E) Quantification of the total number and volume of the LPNP-Au-555-containing phagolysosomes in microglial cells. (F) An illustration of a LPNP-Au by electron microscopy shows a LPNP that contains several 10 nm Au particles (pointed by a white arrowhead). (G–I) Characterization of LPNP-Au nanoparticle accumulation in phagolysosomes by electron microscopy. (G) Microglia are recognized with their densely packed heterochromatin-containing nuclei (asterisk in panel G); four Au-containing phagolysosomes in the microglia distal to the nucleus indicated by the red arrow in panel (G) are shown in high magnification in panel (H); the orange-dotted lines in panel (G) frame the area covered by the microglial soma. (I) One of the 10 nm Au particle-containing phagolysosomes also contains a 15 nm Au particle derived from immunogold labeling for the lysosome marker LAMP1 (pointed by a red arrowhead). (J) Diameter of the Au-containing phagolysosomes in microglia. (K–N) The localization of the siRNA-FAM (green) in the cytosolic compartments of microglial cells with 0.5 h (K, L) or 6 h (M, N) treatment. The lysosomes in the cytosolic compartments are visualized with LysoTracker (red). Higher magnification of the cells pointed by the white arrows in panels (K) or (M) was illustrated in panels (L) or (N). (O) Ratio between the area of coverage of the extra-Lyso siRNA-FAM fluorescence signal and the LysoTracker fluorescence signal in the BV2 cells with 0.5 or 6 h treatment. All the results were presented as means ± SD. The data were analyzed using Student’s *t* test in panel (O). Scale bar: 10 μm in panels (A–C), 100 nm in panel (F), 1 μm in panel (G), 200 nm in panel (H), 100 nm in panel (I), 15 μm in panels (K, M), and 5 μm in panels (L, N).

### Characterization of the Microglial Phagocytotic Capacity

The microglial phagocytotic capacity for LPNPs was quantified by using 3D reconstructed individual microglial cells (Figure 3A–C). The total number and the total volume of LPNP-Au-555-containing phagolysosomes in each microglial cell were 25.6 ± 18.9 and 51.7 ± 41.7 μm^3^, respectively (Figure 3D,E). To confirm the specific uptake of LPNP-Au nanoparticles (Figure 3F) by microglial cells, we further characterized their ultrastructure by electron microscopy. We found that Au nanoparticle-containing phagolysosomes of different sizes were distributed in the cytoplasmic area of microglial cells (Figure 3G,H). Some of the Au-containing phagolysosomes were also labeled by the lysosome marker lysosomal-associated membrane protein 1 (LAMP1), as demonstrated by the colocalization of 10 nm Au particles derived from LPNP-Au and 15 nm Au particles derived from LAMP1 immunogold labeling (Figure 3I). The average diameter of the phagolysosomes in microglia was 441.6 ± 260.8 nm (Figure 3J).

Intriguingly, although confocal fluorescence microscopy did not reveal clear accumulation of LPNP-Au-555-containing phagolysosomes in neurons and astrocytes, electron microscopy indicated a few cells that did not only possess the typical microglial dense highly packed heterochromatin-containing nuclei but also contained Au particles in phagolysosome-like structures (Figure S7A,B). These data indicate that, although microglia are the dominant cell population in the brain that take up LPNPs, some other unidentified non-microglial cells might also take up very few LPNPs.

### Fate Mapping of LPNP-Carried siRNA in the Cytosolic Compartment of BV2 Cells *In Vitro*

Next, we performed fate mapping of the LPNP-carried siRNA in BV2 cells. We encapsulated the carboxyfluorescein (FAM)-labeled siRNA (siRNA-FAM) to the LPNPs and checked the localization of the siRNA-FAM in the cytosolic compartment of the microglial cells. In 0.5 h treated cells, the majority of the siRNA-FAM was colocalized with the lysosomes (labeled with LysoTracker) (Figure 3K,L), whereas in 6 h treated cells, more siRNA-FAM fluorescence signals were found outside of lysosomes (Figure 3M,N). The area of coverage of the siRNA-FAM fluorescence signals outside of lysosomes (Extra-Lyso siRNA-FAM) was significantly higher in the 6 h treated cells than in the 0.5 h treated cells (Figure 3O).

### LPNP-CD11b siRNA Effectively Decreases Microglial CD11b Expression in *In Vitro* and *In Vivo*

**Figure 4:**
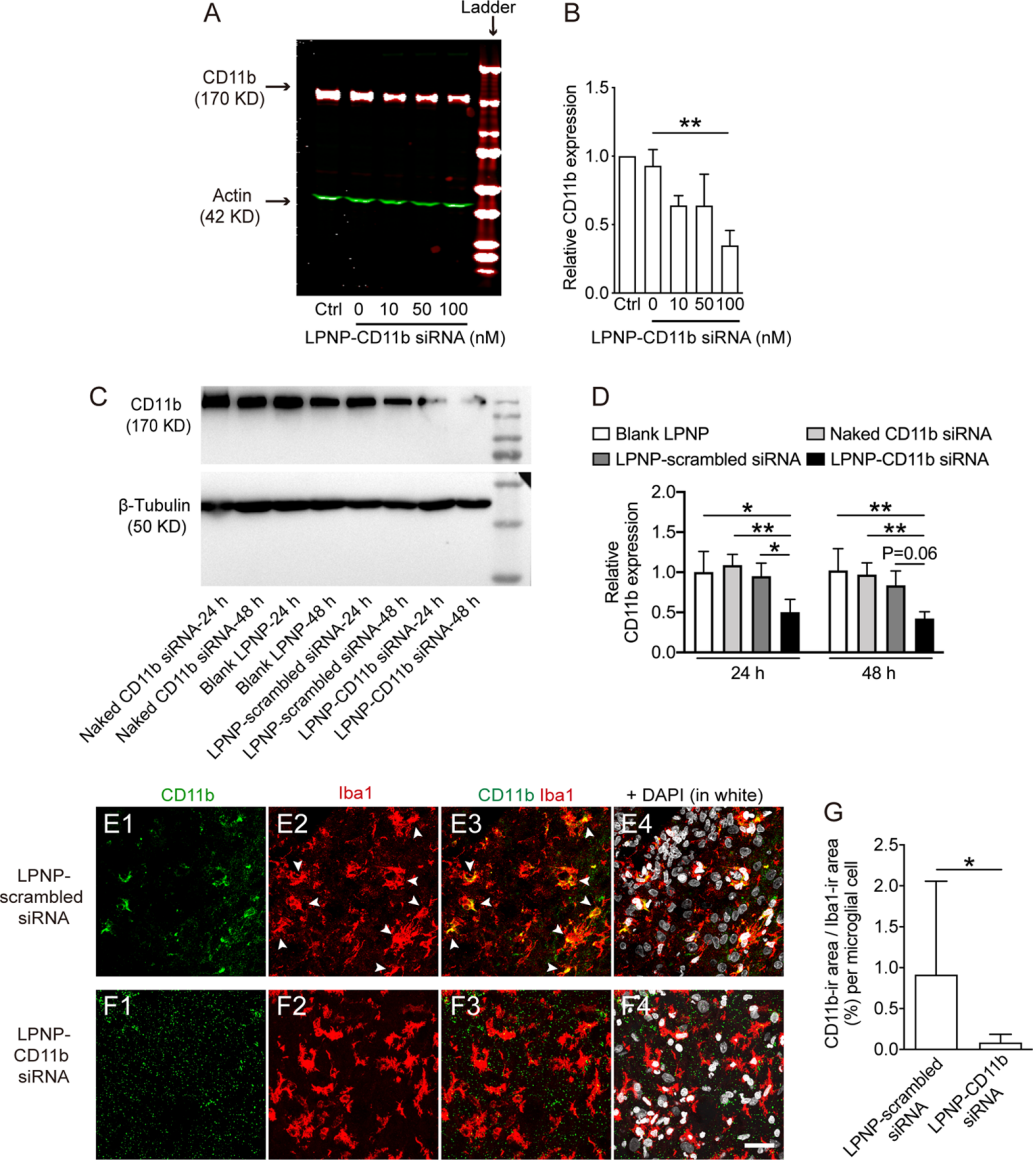
Evaluation of the silencing efficiency by LPNP-CD11b siRNA *in vitro* and *in vivo*. (A, B) After the BV2 cells were treated with LPNP-CD11b siRNA at various siRNA concentrations (0, 10, 50, and 100 nM, with 0 nM representing LPNP-scrambled siRNA (100 nM), *n* = 3), the expression of CD11b was assessed by western blotting. (C, D) In alternative groups, the CD11b protein expressions in the brain tissues isolated from the non-treated control, naked CD11b siRNA, blank LPNP, LPNP-scrambled siRNA, and LPNP-CD11b siRNA injected spots were assessed by western blotting. (E–G) Representative images from the rat hypothalamus after the injection of LPNP-scrambled siRNA (100 nM) or LPNP-CD11b siRNA (100 nM) show colocalization of CD11b-ir in ionized calcium-binding adaptor molecule 1-immunoreactive (Iba1-ir) microglia in the LPNP-scrambled siRNA group (E1–E4), whereas little CD11b-ir is detectable in the LPNP-CD11b siRNA group (F1–F4). The arrowheads in panels (E2, E3) point to Iba1-ir microglia (red) containing CD11b (green) immunoreactivity in the LPNP-scrambled siRNA group. (G) Analysis of the ratio between the coverage area of CD11b and Iba1-ir per microglia. *n* = 3. All data are presented as means ± SD. Data were analyzed using one-way ANOVA in panel (B), two-way ANOVA in panel (D), and Student’s *t* test in panel (G). *p < 0.05, **p < 0.01. Scale bar: 30 μm in panels (E, F).

CD11b is an integrin molecule that is highly expressed on the cell surface of microglial cells in the brain and thus serves as a representative marker for microglia.^33,34^ Therefore, we chose LPNP-CD11b siRNA to evaluate the silencing efficiency of LPNP-siRNA *in vitro* and *in vivo*. First, we treated cultured BV2 cells with LPNP-CD11b siRNA at concentrations of 10, 50, or 100 nM. In microglial cells incubated with 100 nM LPNP-CD11b siRNA for 24 h, the CD11b protein level, as detected by western blotting, was decreased to 34.7% (Figure 4A,B) compared with that in the control group treated with LPNP-scrambled siRNA. We further evaluated CD11b protein expression by immunocytochemical (ICC) staining. The ICC staining results supported the gene silencing capacity of LPNP-CD11b siRNA (Figure S8A–E). We also proved in BV2 cells that the silencing efficiency of CD11b achieved by using the LPNP-CD11b siRNA approach was comparable to the one using the Lipofectamine 3000-delivering method. Both methods had a significant increased silencing efficiency compared to the BV2 cells that received naked CD11b siRNA, blank LPNP, and LPNP-scrambled siRNA (Figure S8F,G).

We then injected the naked CD11b siRNA, blank LPNP, LPNP-scrambled siRNA, or LPNP-CD11b siRNA into the hypothalamus and sacrificed the rats 24 and 48 h following the injections. Twenty-four hours after injection, the CD11b protein expression was significantly downregulated in the LPNP-CD11b siRNA treated group as compared to those that had received blank LPNP, naked CD11b siRNA, or LPNP-scrambled siRNA. These differences in treatment effects remained in the 48 h groups, whereas no effect of time was observed, i.e., no differences between the 24 and 48 h treatments (Figure 4C,D).

To further quantify the CD11b protein expression, in separated groups, we also injected LPNP-CD11b siRNA (100 nM) or LPNP-scrambled siRNA (100 nM) into the rat hypothalamus. The brains were collected by perfusion fixation 20 h after injection and analyzed by immunofluorescence staining to detect the expression of CD11b in microglial cells. We found that CD11b immunoreactivity in the microglial cells surrounding the injection spot was downregulated by 90% compared with the microglial cells in LPNP-scrambled siRNA-injected rats (Figure 4E–G).

### LPNP-TLR4 siRNA Significantly Decreases TLR4 Expression in Microglial Cells *In Vitro* and the Microglial Immune Response *In Vivo*

**Figure 5:**
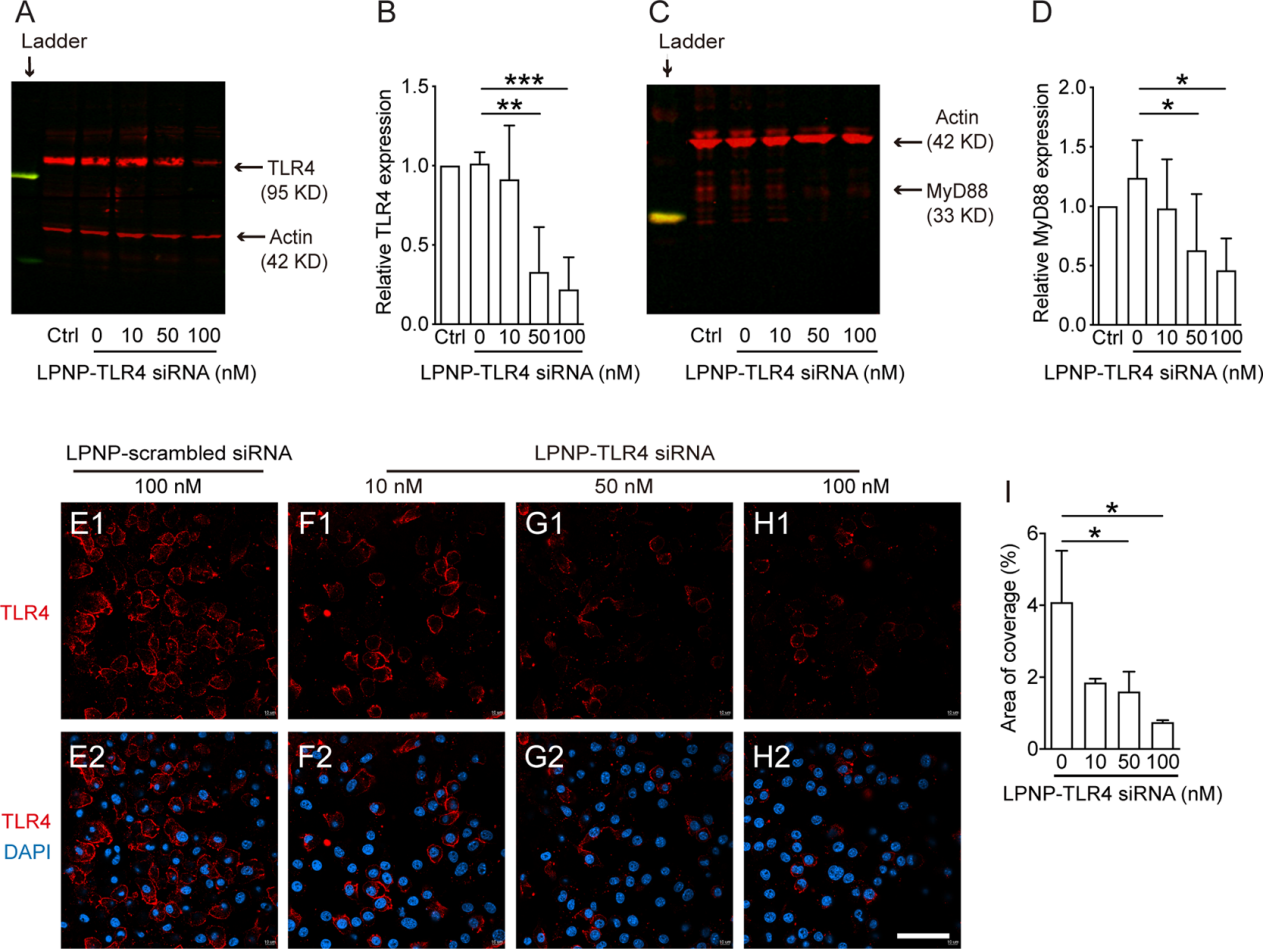
Evaluation of the gene silencing efficiency by LPNP-TLR4 siRNA *in vitro*. (A–D) After the BV2 cells were treated with LPNP-TLR4 siRNA at various siRNA concentrations (0, 10, 50, or 100 nM, with 0 nM representing LPNP-scrambled siRNA (100 nM), *n* = 3), the expression of TLR4 (A, B) and its downstream signaling component MyD88 (C, D) showed significantly lower protein expression in the 50 or 100 nM LPNP-TLR4 siRNA-treated groups, as assessed by western blotting. (E–I) TLR4 protein expression, as assessed by immunocytochemical staining, also showed significant downregulation in the LPNP-TLR4 siRNA-treated groups (*n* = 3). All the data are presented as means ± SD and were analyzed using one-way ANOVA, *p < 0.05; **p < 0.01; ***p < 0.001. Scale bar: 50 μm in panels (E–H).

TLR4 is a key receptor in microglial cells that mediates LPS-stimulated inflammatory responses.^35,36^ Therefore, we used this receptor to provide a functional demonstration of the gene-silencing capacity of LPNP-TLR4 siRNA after an LPS-induced immune challenge. First, we treated cultured BV2 cells with LPNP-TLR4 siRNA at the concentrations of 10, 50, or 100 nM. In microglial cells incubated with 100 nM LPNP-TLR4 siRNA for 24 h, the TLR4 protein level, as detected by western blotting, was decreased to 22.7% (Figure 5A,B) compared with that in the control group treated with LPNP-scrambled siRNA. To examine whether silencing the TLR4 gene expression would also efficiently decrease its downstream signals, we quantified the protein expression of myeloid differentiation primary response 88 (MyD88), a critical adapter protein that mediates signal transduction for TLR4.^37^ We found that the MyD88 protein expression was significantly decreased in the 50 and 100 nM LPNP-TLR4 siRNA-treated microglial cells (Figure 5C,D). The ICC staining results supported the gene silencing capacity of LPNP-TLR4 siRNA *in vitro* (Figure 5E–I). Despite using different TLR4 antibodies, we could not obtain reliable TLR4 immunoreactivity in the rat brain sections; therefore, we did not perform a similar study to detect TLR4 protein expression *in vivo*.

**Figure 6:**
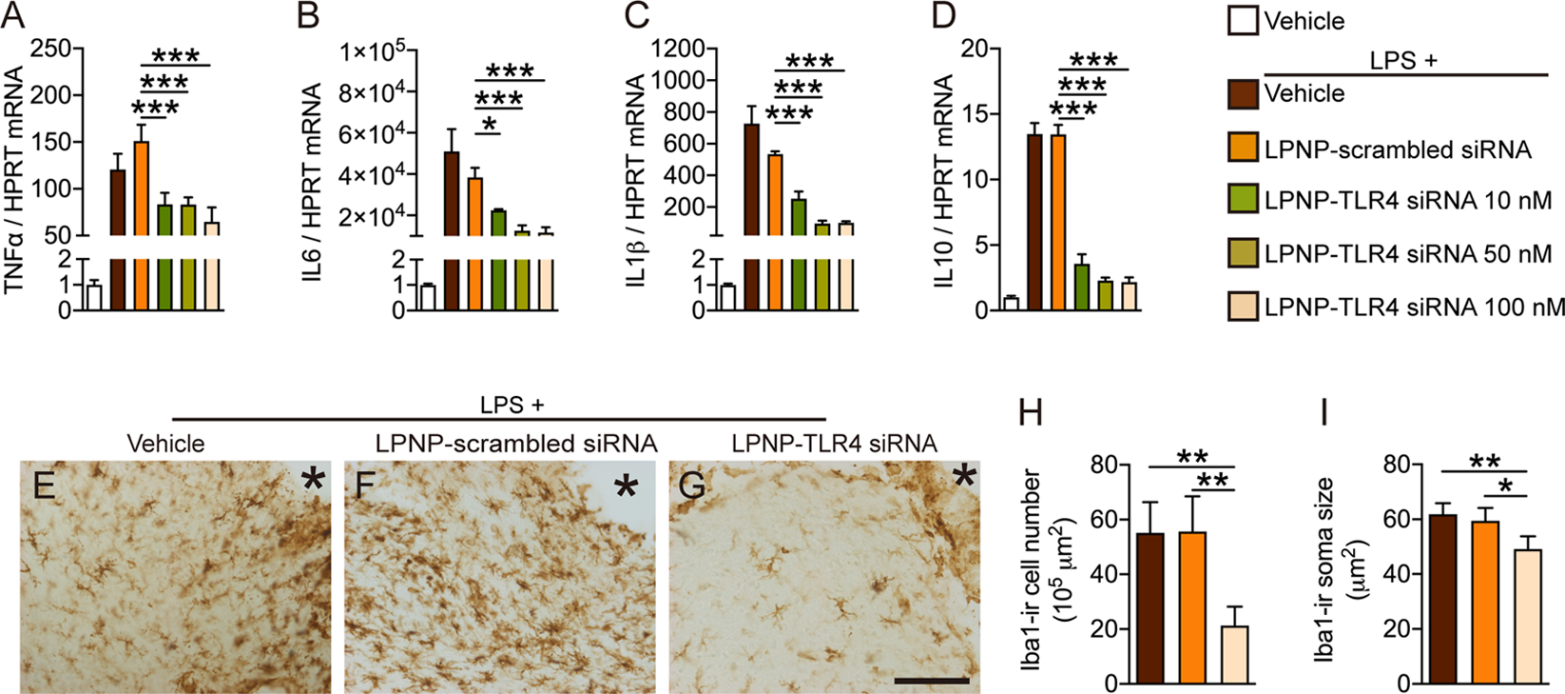
LPNP-TLR4 siRNA downregulates the immune response of microglia following LPS stimulation. (A–D) Gene expression of TNF-α, IL-6, IL-1β, and IL-10 in cultured microglial cells treated for 24 h with LPNP-TLR4 siRNA (*n* = 3) at different concentrations (0, 10, 50, and 100 nM, with 0 nM representing LPNP-scrambled siRNA (100 nM)). Afterward, the cells were treated with LPS (100 ng/mL) for 2 h (in addition to the first PBS group). (E–G) Representative images show Iba1-ir microglial cells near the injection spot (asterisk) at the morphological level in the rat hypothalamus treated with LPNP-TLR4 siRNA 18 h before followed by intravenous LPS (100 μg/kg) 2 h before sacrifice. (H) Quantification of the Iba1-ir microglial cell number and (I) soma size following LPS stimulation (*n* = 4). The data are presented as means ± SD and were analyzed using one-way ANOVA, *p < 0.05; **p < 0.01; ***p < 0.001. Scale bar: 100 μm in panels (E–G).

To demonstrate the functional gene silencing capacity of LPNP-TLR4 siRNA, we performed an *in vitro* study on the microglial inflammatory response to LPS. We incubated cultured BV2 cells with LPNP-TLR4 siRNA at different concentrations (0, 10, 50, or 100 nM) for 24 h followed by incubation with LPS-containing media (100 ng/mL) for 2 h. The gene expressions of TNF-α (Figure 6A), IL-6 (Figure 6B), IL-10 (Figure 6C), and IL-1β (Figure 6D) in the LPNP-TLR4 siRNA-treated cells were significantly lower than those in the cells treated with LPNP-scrambled siRNA, indicating that LPNP-TLR4 siRNA effectively downregulated the microglial immune response to LPS.

We also performed an *in vivo* study on microglial reactivity in response to LPS. We injected LPNP-TLR4 siRNA (100 nM, 1 μL) bilaterally into the rat hypothalamus. Eighteen hours later, the rats received an intravenous infusion of LPS via the right jugular vein. Two hours after the LPS infusion, the rats were sacrificed by perfusion fixation, and the microglia were subjected to Iba1-ir analysis. In the LPNP-TLR4 siRNA-injected group, both the cell number and soma size of the Iba1-ir microglia surrounding the injected spot were decreased significantly (Figure 6E–I) compared with those in the LPS-vehicle group or LPNP-scrambled siRNA group, indicating that LPNP-TLR4 siRNA effectively inhibited microglial activation in response to LPS in the hypothalamus.

## Conclusions and Prospects

In this study, we established a method of using biocompatible and biodegradable nanomaterials to deliver small interfering RNAs (siRNAs) to selectively inhibit target gene expression in microglial cells in a specific brain area. We proved that LPNPs are taken up by microglial cells with a high specificity after local brain infusion, with a limited diffusing zone. Next, we demonstrated that LPNP-CD11b siRNA and LPNP-TLR4 siRNA are effective in silencing CD11b and TLR4 expression, respectively. We also functionally evaluated the immune response of microglia in LPNP-TLR4 siRNA-treated microglial cells both *in vitro* and *in vivo*.

In the current study, we aimed to deliver siRNAs specifically into brain microglial cells. In previous studies, in which mPEG-PLGA nanoparticles were coated with a rabies virus glycoprotein (RVG) 29, it was reported to effectively cross the blood brain barrier, likely via its binding with the nicotinic acetylcholine receptor.^38^ When administered intravenously, the RVG 29-coated mPEG-PLGA nanoparticles could carry compounds to neurons located in the striatum and substantia nigra in the mouse brain.^39^ However, this peripheral administration of RVG29-mPEG-PLGA nanoparticles is not a suitable approach to achieve a specific uptake by microglial cells in the brain, and we therefore decided to choose intracranial stereotactic injections to directly deliver LPNP-siRNA into the hypothalamus of rats. Nevertheless, one of the future research questions is to develop nanocarriers that can cross the BBB efficiently and target microglial cells when delivered systemically, ideally with brain region specificity (like the hypothalamus), to achieve region-specific microglial gene manipulation.

Compared with chemical inhibitors, siRNA is well known for its biocompatibility, stronger suppressive effects, and reduced side effects.^26^ However, siRNA can be rapidly degraded by nucleases in plasma or excreted. Moreover, it can hardly penetrate the cell membrane because of its hydrophilic properties.^26,40,41^ The conjugation of lipid nanoparticles and siRNA can overcome these obstacles and has been proven to be a safe and effective *in vivo* siRNA delivery approach.^26,41^ We exploited these advantages and further proved that this type of LPNP-siRNA is effective in modifying brain microglial function.

In the current study, by using fluorescence and electron microscopy, we demonstrated that, as siRNA carriers, LPNPs are predominantly taken up by microglial cells in the phagolysosomes. We showed *in vitro* that the LPNP-delivered siRNA could be released from the lysosomes into the cytosolic compartment in the microglial cells, as also described previously.^27^ Therefore, we assumed, with a similar endocytic recycling mechanism in generic cells, that the CD11b siRNAs and TLR4 siRNA in the microglial cells in the rat brain also escaped from the phagolysosome efficiently. Nevertheless, due to the digestive function of lysosomes, the degradation of NPs is unavoidable. Thus, question remains how to modify nanocarriers to enhance the escape of nanoparticle-delivered drugs or siRNA from endosomes or lysosomes.^42,43^

Another important aspect is the potential of the nanomaterial *per se* to cause adverse effects in microglial cells. Therefore, all materials used in this study to synthesize the LPNPs are widely used for scientific research or clinic trials. Although potential safety risks are associated with any type of NP,^28^ there is extensive literature showing that PLGA NPs and lipid NPs represent a safe means of drug delivery.^44^ In our *in vitro* studies, we found no evidence for LPNPs or LPNP-scrambled siRNA-induced cytotoxicity or an immune response in cultured microglial cells. Additionally, in the *in vivo* studies, we found no evidence that the LPNPs caused activation of microglia, in addition to those microglial cells directly injured at the injection spot. Furthermore, LPNP-siRNA-injected rats did not differ from the control animals with respect to food intake, body weight, or general grooming behavior (data not shown). Thus, LPNP-siRNA is a safe tool for manipulating microglial function in the brain and for treating microglia-related disorders.

TLR4 is highly expressed in microglial cells.^45^ Upon microbial stimulation, TLR4 initiates the activation of innate immune responses.^46^ It has been shown that, in high fat diet-induced obese animals, TLR4 signaling in the hypothalamus is activated by saturated fatty acids and that this TLR4 activation and its associated cascade inflammatory responses in the microglial cells are required for the induction of leptin resistance and obesity.^23,24^ Thus, to treat obesity, one of the targets in the brain is the microglial TLR4 pathway in the hypothalamus. Therefore, we expect that, in high fat diet-induced obese animals, chronic delivery of LPNP-TLR4 siRNA will effectively reduce the microglial activation and prevent the animals from the diet-induced obesity, although in the current study, we have only tested the knocking down efficiency of LPNP-TLR4 siRNA by LPS. To this end, once the obstacle of delivering LPNP-siRNA across the blood brain barrier is conquered, the LPNP-siRNA approach we established in the current study will serve not only as a research tool but also a potential therapeutic approach to combat metabolic syndromes.

It is well known that microglial dysfunctions are involved in Alzheimer’s disease (AD).^47^ AD is characterized by the accumulation of extracellular plaques of toxic amyloid-beta (Aβ), which puts high demands on effective clearance by the neighboring microglial cells.^48^ Evidence is accumulating that long-term exposure to Aβ induces chronic reactive microgliosis, characterized by increased inflammatory factors and comprised phagocytosis.^49,50^ Thus, another potential application of our method is to normalize the microglial function in AD. This includes, on one hand, to suppress the detrimental pro-inflammatory factors and/or to enhance the beneficial anti-inflammatory protective functions and, on the other hand, to enhance the phagocytic capacity. These can be achieved by coating different siRNA and/or packing different compounds that target inflammatory and phagocytic pathways with the LPNPs.

## Experimental Section

### Preparation of Lipid-Polymer Hybrid Nanoparticles

The copolymer nanoparticles were synthesized by the double-emulsion (W/O/W) method as previously described.^25,51,52^ First, 20 mg of mPEG-PLGA (mPEG: MW 5000; PLGA: MW 15,000, molar ratio of d,l-lactic to glycolic acid = 75:25) was dissolved in 1 mL of dichloromethane; thereafter, 0.2 mL of water was added. The mixture was emulsified by sonication for 5 min followed by the addition of 2 mL of 2% polyvinyl alcohol (PVA; 363146; Sigma-Aldrich) with 10 mg of ε-polylysine (EPL; FP14985; Carbosynth). The emulsion was then sonicated for another 5 min, mixed with 10 mL of 0.6% PVA, and stirred for 10 min at room temperature. Next, dichloromethane was removed by a vacuum rotary evaporator and the copolymer nanoparticles were washed twice with distilled water by centrifugation at 13,000 rpm for 10 min at room temperature. Phospholipids (20 mg, 18 mg of DSPC (850365P), 2 mg of DSPE-mPEG_2000_ (F2017000595)), and 4 mg of a cholesterol (C8667; Sigma-Aldrich) mixture were dissolved in dichloromethane, and then, a lipid film was formed in a round-bottomed flask using the vacuum rotary evaporator. Following the addition of the polymer emulsion, the ε-polylysine-modified copolymer nanoparticles were coated with a lipid film during sonication for 5 min. The lipid-polymer hybrid nanoparticles (LPNPs) were washed twice with distilled water by centrifugation at 13,000 rpm for 10 min at room temperature.

### Generation of LPNP-Rhodamine B, LPNP-Au-555, and LPNP-siRNA

To generate LPNP-rhodamine B or LPNP-Au-555, the “0.2 mL of water” step described above under the subheading “Preparation of Lipid-Polymer Hybrid Nanoparticles” was replaced by rhodamine B solution or Alexa-555-Au NP (prepared by conjugating Alexa-555 with 10 nm gold particles^953^). The subsequent steps were the same as described above in the Preparation of Lipid-Polymer Hybrid Nanoparticles section. To generate LPNP-siRNA, we mixed the siRNA solution with the washed EPL-modified copolymer nanoparticles as described above to absorb the siRNAs on the surface of the nanoparticles. The siRNAs were purchased from Horizon-Inspired Cell Solutions (Horizon Discovery, SMSRTpool) (for detailed siRNA sequences, see the Supporting Information). The concentration of siRNA used was 100 nM, and the concentration of mPEG-PLGA was 5.33 μg/mL. The ratio (w:w) of siRNA:EPL:mPEG-PLGA:lipids was 1:2:4:4. The coating of the lipid film was the same as described above in the Preparation of Lipid-Polymer Hybrid Nanoparticles section. All the materials and reagents for LPNP-siRNA synthesis were nuclease-free.

### Characterization of Nanoparticles

To prove the sequential package of the hybridized nanoparticles, we tested the surface charge of the nanoparticles after adding different amounts of EPL, RNA, and lipids. The size distribution and surface charge were examined using a ZetaSizer Nano series Nano-ZS system (Malvern Instruments Ltd., Malvern, UK) equipped with a He-Ne laser beam at a wavelength of 633 nm and a fixed scattering angle of 90°. The nanoparticles were diluted in distilled water and determined at 25 °C. For morphology characterization, LPNP-siRNA and LPNP-Au nanoparticles were negatively stained with 2% uranyl acetate solution and deposited on a carbon-coated copper grid, and images were acquired using transmission electron microscopy (TEM; JEM-200CX; Jeol Ltd., Japan). The RNA encapsulation efficiency of the LPNPs was calculated by the following equation: encapsulation efficiency = (*A* – *B*)/*A* × 100%, where *A* is the initial amount of RNA put in the system and *B* is the amount of RNA in the supernatant after centrifuging the mixture of RNA solution with the EPL-modified copolymer nanoparticles. The amount of RNA was determined by a NanoDrop device (ND2000, Thermo NanoDrop).

### Uptake and Degradation of LPNPs by Microglial Cells

BV2 cells were seeded into an eight-well chamber (Thermo Fisher Scientific; 155411PK) and cultured to 70–80% confluence. To observe the uptake, the cells were treated with LPNP-RhoB for different times (0.5, 1, 4, and 24 h) and washed with PBS; to observe the degradation, we first cultured the cells with LPNP-RhoB for different durations (0.5, 1, 2, 3, and 4 h), the medium was then replaced with a fresh medium, and the cells were cultured for another 16 h. The fluorescence images were acquired using a Leica TCS SP5 inverted confocal microscope.

### Localization of LPNP-siRNA-FAM in Microglial Cells

BV2 cells were seeded onto a 29 mm glass bottom dish (D29-20-1-N, Cellvis) at a density of 2 × 10^5^ cells/well. Cells were then treated by the carboxyfluorescein-labeled siRNA (FAM-siRNA) and encapsulated LPNPs (LPNP-siRNA-FAM) for 0.5 h (*n* = 4) or 6 h (*n* = 4), respectively. The cells were then washed three times with PBS. Lysosomes were labeled by LysoTracker Red (C1046, Beyotime Biotechnology) according to the manufacturer’s instructions. Cells were then briefly fixed with 1% paraformaldehyde for 10 min and washed three times with PBS. Afterward, cells were observed with a confocal microscope (STELLARIS 5, Leica) at 63× magnification. To quantify the siRNA-FAM fluorescence signals outside of lysosomes, the area of coverage of the intra-Lyso siRNA (yellow signals), the total siRNA (all green signals, including intra-Lyso siRNA and extra-Lyso siRNA (green signals)), and the total lysosomes (red LysoTacker signals) were quantified. The percentage of extra-Lyso siRNA per cell was calculated as “100 × (green – yellow)/red”, i.e., 100 × (total siRNA – intra-Lyso siRNA)/LysoTracker.

### Detection of the Silencing Efficiency of LPNP-siRNA *In Vitro* by Western Blotting and Immunocytochemistry

For LPNP-siRNA treatment, BV2 cells were seeded at a density of 5 × 10^5^ cells/well in six-well plates. When the cells were 80% confluent, LPNP-TLR4 siRNA or LPNP-CD11b siRNA at different concentrations (0, 10, 50, and 100 nM) was incubated with the cells for 24 h. An additional experiment was performed to compare the silencing efficiency in BV2 cells treated with LPNP-CD11b siRNA (LPNP, 0.014 mg/mL; CD11b siRNA, 100 nM), naked CD11b siRNA (100 nM), blank LPNPs (0.014 mg/mL), LPNP-scrambled siRNA (LPNP, 0.014 mg/mL; scrambled siRNA, 100 nM), or Lipofectamine 3000-delivered CD11b siRNA (100 nM CD11b siRNA, L3000015, Thermo Fisher Scientific), for 48 h. TLR4 or CD11b protein expression was analyzed using western blotting and/or immunocytochemistry (ICC).

#### Western Blotting

After 24 h of treatment, adherent cells were washed with ice-cold PBS and homogenized in lysis buffer. The protein concentration in the supernatant was determined using a BCA protein assay kit (Thermo Fisher Scientific), and proteins were resolved on 10% SDS/PAGE gels. After transfer to 0.45 μm PVDF membranes, the membranes were blocked for 1 h in blocking buffer and then incubated overnight at 4 °C with the primary TLR4 or CD11b antibody (TLR4: ab13556, Abcam; CD11b: ab133357, Abcam; MyD88: ab2064, Abcam; Actin: sc-1616, Santa Cruz Biotechnology) in the same buffer. The blots were then washed in TBS-T (TBS buffer containing 0.1% Tween-20), incubated for 1 h with fluorescently labeled secondary antibodies, and detected using Odyssey Imaging Systems.

#### Immunocytochemistry

After treatment with LPNP-TLR4 siRNA or LPNP-CD11b siRNA at different concentrations in the Lab-Tek chamber (155411PK734-2062 vwr), the microglial cells were washed with PBS and fixed with 1% paraformaldehyde solution for 10 min. After rinsing the cells with TBS, the TLR4 (Abcam, ab13556, 1:200) or CD11b (Abcam, ab133357, 1:200) primary antibody was incubated (in SUMI buffer (2.5 mg/mL gelatin, 0.5% Triton, dissolved in TBS)) with the cells overnight at 4 °C. After rinsing with TBS, the secondary antibody (biotinylated anti-rabbit, Vector Lab, BA1100, 1:400) was incubated with the cells for 1 h at room temperature. The cells were washed with TBS and incubated with streptavidin Alexa Fluorophore (for TLR4: streptavidin Alexa Fluorophore 594, Jackson ImmunoResearch, 016-580-084, 1:300; for CD11b: streptavidin Alexa Fluorophore 488, Invitrogen, s32354, 1:300) for 1 h at room temperature. After washing with TBS, the cells were incubated with DAPI (Sigma-Aldrich; 1:1000) for 10 min. Following washes in TBS, the fluorescence signals in cells were detected using a Leica TCS SP5 inverted confocal microscope.

### Response of LPNP-TLR4 siRNA- and LPNP-CD11b siRNA-Treated Microglial Cells to LPS Immune Challenge *In Vitro*

BV2 cells were treated with LPNP-TLR4 siRNA at different concentrations (0, 10, 50, and 100 nM, with 0 nM representing LPNP-scrambled siRNA) for 24 h. Next, the cells (besides the first PBS group) were treated with LPS (100 ng/mL; Alpha Diagnostic; LPS16-1) for 2 h. Total RNA was isolated from the cells using an RNA isolating kit (BIO-52073). The samples were subjected to the reverse-transcription polymerase chain reaction system (Roche, 04896866001). One milligram of cDNA was quantified by real-time PCR using primer pairs with an SYBR Green PCR Master Mix (Roche LightCycler 480). Each sample was analyzed in triplicate. The gene expressions of TNF-α, IL-6, IL-10, and IL-1β were measured by RT-PCR using HPRT as a housekeeping gene (Supporting information, Table S1).

### Surgery for Brain Infusion of LPNPs and Systemic Infusion of LPS

All the animal studies were approved by the Animal Ethics Committee of the Royal Dutch Academy of Arts and Sciences (KNAW; Amsterdam) and were performed according to the guidelines on animal experimentation of the Netherlands Institute for Neuroscience (NIN, Amsterdam). Male Wistar rats (Charles River, Germany) were housed on a 12 h light/12 h dark cycle (lights on at 7:00 am) at 22 ± 2 °C with access to food and water *ad libitum*.

For the infusion of LPNP-RhoB or LPNP-Au-555 into the hypothalamus or infusion of LPNP-siRNA into the hypothalamus in combination with the infusion of LPS into the general circulation, the rats (body weight, 300–350 g) were subjected to surgery after receiving anesthesia with i.m. 80 mg/kg ketamine (Eurovet Animal Health, Bladel, Netherlands) and 8 mg/kg Rompun (Xylazine; Bayer Health Care, Mijdrecht, Netherlands). For the infusion of LPNP-RhoB or LPNP-Au-555 using a standard Kopf stereotaxic apparatus, 1 μL of LPNP-RhoB or LPNP-Au-555 (4 O.D.) was injected using a Hamilton Microliter syringe (7000 series; Hamilton) bilaterally into the lateral hypothalamus (for LPNP-RhoB) or the mediobasal hypothalamus (for LPNP-Au-555), with the coordinates adapted from the atlas of Paxinos and Watson (for LPNP-RhoB: anteroposterior: −2.8 mm; lateral: 1.4 mm; ventral: −9.0 mm; for LPNP-Au-555: anteroposterior: −2.8 mm; lateral: 0.6 mm; ventral: −9.8 mm).^53^ The rats were sacrificed 4 h (for LPNP-RhoB) or 24 h (for LPNP-Au-555) later by perfusion fixation (0.9% saline flush followed by 4% paraformaldehyde). For immunohistochemical and immunofluorescence staining, the brains were collected and went through 16 h post-fixation with 4% paraformaldehyde. For electron microscopy (see below), hypothalamic tissue blocks of 1.0 mm^3^ were dissected under a fluorescence microscope to define the LNPN-Au-555 diffusion area. The tissue was then further fixed in 0.2% glutaraldehyde for 24 h.

For the infusion of LPNP-siRNA, brain infusion probes (Guide Cannula, P1 Technologies) were placed into the mediobasal hypothalamus with the coordinates adapted from the atlas of Paxinos and Watson (anteroposterior: −2.8 mm; lateral: 2.0 mm; angle: 8°; ventral: −10 mm).^45^ For infusion of LPS into the general circulation, a silicone catheter was inserted into the right jugular vein for i.v. infusions of LPS. The catheter and probes were fixed on top of the head and secured with dental cement. After recovery from the surgery, five experiments were performed: (i) Infusion of LPNP versus vehicle into the hypothalamus to examine the effects of LPNP on microglial activity *in vivo.* Twenty hours following the administration of LPNP or vehicle, the rats were sacrificed by perfusion fixation, and the brains were collected. (ii) Infusion of LPNP-RhoB or LPNP-Au-555 into the hypothalamus to trace the fate of LPNPs *in vivo*. Four hours following administration of LPNP-RhoB and 24 h following administration of LPNP-Au-555, rats were sacrificed by perfusion fixation, and the brains were collected for immunofluorescence imaging and electron microscopy. (iii) Infusion of LPNP-CD11b siRNA into the hypothalamus to quantify the silencing efficiency of LPNP-CD11b siRNA *in vivo* by immunohistochemical and immunofluorescence staining*.* Twenty hours following the administration of LPNP-scrambled siRNA or LPNP-CD11b siRNA, the rats were sacrificed by perfusion fixation, and the brains were collected. (iv) Infusion of LPNP-CD11b siRNA into the hypothalamus to quantify the silencing efficiency of LPNP-CD11b siRNA *in vivo* by western blotting. The rats were sacrificed 24 or 48 h following the administration of naked CD11b siRNA (100 nM), blank LPNPs (14.4 μg/mL), LPNP-scrambled siRNA (LPNP, 14.4 μg/mL; scrambled siRNA, 100 nM), and LPNP-CD11b siRNA (LPNP, 14.4 μg/mL; 100 nM CD11b siRNA). The whole brain was isolated and placed on stainless-steel rodent brain matrices (Harvard Apparatus) to cut out the coronal brain slice (1 mm thickness) that includes the injection spot. Using the most intensive Rhodamine B fluorescence as the control spot, the brain slice was further microdissected within a ≈300 μm radius area surrounding the control spot, under a fluorescence microscope with a surgery scalpel (all steps were performed at 4 °C). The brain region surrounding the injection spot was collected and saved at −80 °C. (v) Infusion of LPNP-TLR4 siRNA into the hypothalamus followed by the infusion of LPS into the general circulation to functionally examine the efficiency of LPNP-TLR4 siRNA to inhibit the microglial immune response to LPS. Eighteen hours after the infusion of saline, LPNP-scrambled siRNA, or LPNP-TLR4 siRNA via the brain probes, the rats received an intravenous infusion of LPS (100 μg/kg). Two hours later, the rats were sacrificed by perfusion fixation, and the brains were collected for “immunohistochemical and immunofluorescence staining”.

### Immunohistochemical and Immunofluorescence Staining

All rat brains that had received nanoparticle infusions were collected by perfusion fixation with 4% paraformaldehyde and post-fixation for another 16 h. The brains were then equilibrated with 30% sucrose, and coronal sections (35 μm) were cut on a cryostat. From the sections in each brain, we selected four to six sections around the injection spots (two to three sections on each side of the injections spot) based on the fact that the diffusion area of the nanoparticles is ≈300 μm around the injection spot. We noticed that not all the sections are intact at the level of injection, due to the mechanical damage caused by the infusion needle, or the brain infusion probes. Nevertheless, for all the staining sections, we have chosen the best quality sections for each brain.

For the detection of LPNP-RhoB in the brain (Figure S4), two sections were used for immune-staining of Iba1 (Figure S4A,B, to visualize microglia), two consecutive sections were used for immune-staining of GFAP (Figure S4C, to visualize astrocytes), and two other consecutive sections were used for immune-staining of orexin (Figure S4D, to visualize orexin-expressing neurons). The sections were incubated at 4 °C overnight with the Iba1 primary antibody (Synaptic Systems; no. 234003; 1:400), GFAP primary antibody (DAKO; Z0334; 1:400), or orexin primary antibody (Abcam; Ab6214; 1:1000) followed by the secondary antibody (Vector Lab; BA1100; 1:400) and streptavidin Alexa Fluorophore 488 (Invitrogen; s32354; 1:300) for 1 h.

For the detection of LPNP-Au-555 in Iba1-ir microglial cells (Figures 2 and 3), two sections per rat were used for immune-staining of Iba1 and counter-staining of NeuroTrace-640 and two consecutive sections were used for immune-staining of Iba1 and GFAP. The sections were incubated at 4 °C overnight with the Iba1 primary antibody (Synaptic Systems; no. 234003; 1:400) followed by the secondary antibody (Vector Lab; BA1100; 1:400) and streptavidin Alexa Fluorophore 488 (Invitrogen; s32354; 1:300) for 1 h. These sections were then counterstained with NeuroTrace-640 (a fluorescence Nissl staining; Thermo Fisher Scientific; N-21483; 1:200). To visualize the colocalization of LPNP-Au-555 with Iba1 and GFAP, the sections were incubated at 4 °C overnight with Iba1 (Abcam; Ab107159; 1:400) and GFAP (DAKO; Z0334; 1:400) primary antibodies followed by the biotinylated secondary antibody (for Iba1: Vector Lab; BA9500; 1:400) for 1 h. After rinsing with TBS, the brain sections were incubated with streptavidin Alexa Fluorophore 488 (for Iba1: Invitrogen; s32354; 1:300) and Alexa Fluor 647 (for GFAP: Invitrogen; 406414).

For the costaining of Iba1 and CD11b (in Figure 4E,F), two to three sections per brain were used. The sections were incubated at 4 °C overnight with Iba1 (Abcam; Ab107159; 1:400) and CD11b (Abcam; ab133357; 1:200) primary antibodies followed by the biotinylated secondary antibody (Vector Lab; BA1100; 1:400) and then coincubation with streptavidin Alexa Fluorophore 488 (Invitrogen; s32354, for CD11b) and Alexa Fluor 594 (Thermo Fisher Scientific; A11058; 1:200) for 1 h. All brain sections for immunofluorescence staining were costained with DAPI (1:2000) for 10 min at the end of the staining procedure and rinsed in TBS before mounting.

All the immunofluorescence staining sections were dried and covered with Antifade Mounting Media (VECTASHIELD; Vector Labs). The fluorescence signals in sections were detected using a Leica TCS SP8 confocal microscope.

For single immunohistochemical staining for Iba1 (in Figure 6E–G and Figure S5), two to three sections per brain were used. The sections were incubated with the Iba1 primary antibody (Synaptic Systems; no. 234003; 1:2000) at 4 °C overnight. The sections were rinsed and incubated in the biotinylated secondary antibody (Vector Lab; BA1100; 1:400) and avidin-biotin complex (Vector Laboratories, Inc., Burlingame, CA; 1:800). The reaction product was visualized by incubation in 0.5% diaminobenzidine with 0.01% hydrogen peroxide. Afterward, the sections were mounted, ethanol-dehydrated, xylene-cleared, and covered with a coverslip (Entelan). Images were captured by light microscopy with a Zeiss Axioplan 2 Evolution MP camera.

### Western Blotting for the LPNP-CD11b siRNA-Administered Brains

The brain samples were homogenized in RIPA lysis buffer (Solarbio, R0010) containing protease inhibitor tablets (4693159001, Roche) using an automatic bead-based homogenizer (SAIERTE, China). The protein was collected from the supernatant after centrifugation at 12,000 rpm for 15 min, the protein concentration was determined using the BCA protein assay kit (Thermo Fisher Scientific), and proteins were resolved on 10% SDS/PAGE gels. After transfer to 0.45 μm PVDF membranes, the membranes were blocked for 1 h in blocking buffer and then incubated overnight at 4 °C with the primary antibody (CD11b: ab133357, Abcam; β-tubulin: A01030, Abbkine) in the same buffer. The blots were then washed in TBS-T (TBS buffer containing 0.1% Tween-20), incubated for 1 h with secondary antibodies (Goat Anti-Rabbit IgG (H&L)-HRP: BE0101, EASYBIO; Goat Anti-Mouse IgG (H&L)-HRP: BE0102, EASYBIO), and detected using a Tanon 5200 imaging system.

### Image Analysis

The relative fluorescence intensity of TLR4-ir and CD11b-ir *in vitro* was measured by FiJi (an ImageJ distribution). Images for the quantification of LPNP-Au-555 in Iba1-ir microglial cells *in vivo* were taken with a 15 μm z-stack (0.05 μm per stack). Images for the quantification of CD11b-ir in Iba1-ir microglial cells *in vivo* were taken using 5 μm z-stack. In Figure 2, in four sections with Iba1-ir (costained with NeuroTrace or GFAP), the total number of Iba1-ir microglia within the LPNP-Au-555 diffusion area surrounding the injection spot was manually counted, microglia without LPNP-Au-containing phagolysosomes among the total Iba1-ir microglia were also quantified, and the percentage was calculated. In Figure 3, the quantification of the LPNP-Au-containing phagolysosomes in individual microglial cells *in vivo* was performed using Imaris software (version 9.5.1; Oxford Instruments, Abingdon, UK). To quantify the total volume of LPNP-Au-555-containing phagolysosomes per Iba1-ir microglial cell, 3D reconstructed images were generated with the z-stack raw image data, the LPNP-Au-555 signal was then used to generate surfaces with fixed threshold settings, and the number and the volume of LPNP-Au-555-containing phagolysosomes were measured. The quantification of CD11b-ir in Iba1-ir microglial cells *in vivo* was obtained by dividing the total area of coverage of green fluorescence signals (CD11b-ir) by the total area of coverage of red fluorescence signals (Iba1-ir). Light microscopy images of immunohistochemical staining for Iba1 were acquired using a Zeiss Axioplan 2 Evolution MP camera. The cell number and area of coverage were analyzed by FiJi, and data obtained from different sections per rat were then averaged for each rat for final statistical analysis.

### Electron Microscopy with Brain Tissues

Hypothalamic tissue blocks of 1.0 mm^3^ were dissected and carefully washed in 0.1 M PHEM washing buffer. The samples were embedded in an increasing percentage of gelatin in PC buffer at 37 °C and incubated overnight in 2.3 M sucrose. After plunge freezing in liquid nitrogen, semi- and ultrathin sections were cut using a Leica ultramicrotome (UC6) and a cryo-EM diamond knife. Semi-thin 150–250 nm thin sections were analyzed using a wide-field fluorescence microscope (Leica DM6), and several 60 nm ultrathin sections were cut and then collected on copper grids. The grids were immunogold-labeled using the LAMP1 antibody (CD107a; BD Pharmingen) and protein A conjugated to 15 nm gold (Utrecht University) and finally contrasted with uranyl acetate in tylose before imaging. Images were obtained using an FEI Tecnai T12 transmission electron microscope at 120 kV and a Velata and Xarosa digital camera (RADIUS software). Microglia, which could be identified by dense and highly packed heterochromatin-containing nuclei, were imaged at various magnifications for posterior analysis.^54^

### Statistical Analysis

All the results were presented as means ± SD. Two-tailed Student’s *t* test and one-way or two-way ANOVA followed by post hoc analysis were used to test for differences between individual experimental groups. Differences between groups were considered statistically significant when two-sided *p* < 0.05. Statistics were calculated using IBM SPSS version 22 or GraphPad Prism 7 software.

## Supporting Information Available

Manufacturer of mPEG-PLGA and Dulbecco’s modified Eagle’s medium (DMEM) and fetal bovine serum, sequences of all the siRNA used in this work, sequences of the primers used for RT-PCR (Supporting Information, Table S1), cell viability of BV2 cells treated with LPNPs at different concentrations for 24 h (Figure S1A), pro-inflammatory cytokine gene expression in BV2 cells treated with different LPNP concentrations for 24 h (Figure S1B–D), accumulation of LPNP-siRNA in BV2 cells upon different durations of treatment *in vitro* (Figure S2), accumulation and degradation of LPNP-RhoB in BV2 cells (Figure S3), specific accumulation of LPNP-RhoB in microglial cells in the rat hypothalamus (Figure S4), the response of microglial cells following LPNP injection in the brain (Figure S5), characterization of the size and the zeta potential of LPNP-Au nanoparticles (Figure S6), characterization of the LPNP-Au nanoparticle accumulation in phagolysosomes by electron microscopy (Figure S7), and evaluation of the silencing efficiency of LPNP-CD11b siRNA by western blotting and by immunocytochemistry *in vitro* (Figure S8) (PDF)

## Abbreviations

TLR4: Toll-like receptor 4
CD11b: differentiation molecule 11b
MyD88: myeloid differentiation factor 88
siRNAs: small interfering RNAs
LPS: lipopolysaccharide
CSF1R: colony-stimulating factor-1
NPs: nanoparticles
TEM: transmission electron microscopy
Iba1: ionized calcium-binding adaptor molecule 1
GFAP: glial fibrillary acidic protein
LAMP1: lysosomal-associated membrane protein 1
ICC: immunocytochemical
MyD88: myeloid differentiation primary response 88
RVG: rabies virus glycoprotein
