# Supplementary material for: Specific Silencing of Microglial Gene Expression in the Rat Brain by Nanoparticle-Based Small Interfering RNA Delivery
Source: ACS Appl Mater Interfaces. 2022 Jan 18;14(4):5066–79. doi: 10.1021/acsami.1c22434 (PMC8815040; doi:10.1021/acsami.1c22434)
Supplement: Supplementary file 1 — am1c22434_si_001.pdf [file am1c22434_si_001.pdf]

**Supporting Information**

# Specific silencing of microglial gene expression in the rat brain by nanoparticle-based siRNA delivery

Shanshan Guo[#], Fernando Cázarez-Márquez[#], Han Jiao, Ewout Foppen, Nikita L. Korpel, Anita E. Grootemaat, Nalan Liv, Yuanqing Gao, Nicole van der Wel, Bing Zhou, Guangjun Nie, Chun-Xia Yi*

[#] These authors contributed equally to this study.

Corresponding author: Chun-Xia Yi, E-mail: c.yi@amsterdamumc.nl.

Shanshan Guo, Fernando Cázarez-Márquez, Han Jiao, Ewout Foppen, Nikita L. Korpel, Chun-Xia Yi
Department of Endocrinology and Metabolism, Laboratory of Endocrinology, Amsterdam Gastroenterology Endocrinology Metabolism Research Institute, Amsterdam University Medical Centre (UMC), location AMC, University of Amsterdam. Amsterdam, The Netherlands.

Han Jiao, Yuanqing Gao
Key Laboratory of Cardiovascular and Cerebrovascular Medicine, School of Pharmacy, Nanjing Medical University, Nanjing, China.

Fernando Cázarez-Márquez, Ewout Foppen, Nikita L. Korpel
Netherlands Institute for Neuroscience, an Institute of the Royal Netherlands Academy of Arts and Sciences, Amsterdam, The Netherlands.

Anita E. Grootemaat, Nicole van der Wel
Cellular Imaging Core Facility, Amsterdam University Medical Centre (UMC), location AMC, University of Amsterdam, The Netherlands.

Nalan Liv
Section Cell Biology, Center for Molecular Medicine, University Medical Center Utrecht, Utrecht, The Netherlands.

Bing Zhou
Institute of Synthetic Biology, Shenzhen Institutes of Advanced Technology, Chinese Academy of Sciences, Shenzhen, China.

Guangjun Nie

CAS Key Laboratory for Biomedical Effects of Nanomaterials and Nanosafety, National Center for Nanoscience and Technology, Beijing, China.

**Supporting Information**

**Materials and Methods**

Poly (lactic-co-glycolic acid) (PLGA, molar ratio of D, L-lactic to glycolic acid, 75:25), monomethoxypoly (ethylene glycol) (mPEG) was purchased from Jinan Daigang Biotechnology Co. Dulbecco's modified Eagle's medium (DMEM) and fetal bovine serum were purchased from Gibco BRL (Grand Island, NY, USA). All the chemicals used were of analytical reagent quality.

**SMSRTpool siRNA sequences:**

Scrambled siRNA (Dharmacon™, SO-2830407G):

UGGUUUACAUGUCGACUAA, UGGUUUACAUGUUGUGUGA,

UGGUUUACAUGUUUUCUGA, UGGUUUACAUGUUUUCCUA.

SIGENOME Rat Tlr4 (M-090819-01-0005, 29260) siRNA - SMARTpool,

UGACAAACCUAGAACAUGU, GAAAUGCCAUGAGCUUUAG,

GUUGGUGUAUCUUUGAAUA, GGCAGCAGGUCGAAUUGUA

siGENOME Mouse Tlr4 (M-047487-01-0005, 21898) siRNA - SMARTpool:

GCAUAGAGGUAGUUCCUAA, GGAAUUGUAUCGCCUUCUU,

CUUGACAUCUCUUAUACUA, CCUAUUGGACAGCUUAUAA

siGENOME Rat Itgam (CD11b) (M-087794-01-0005,25021) siRNA - SMARTpool:

GGAGAACACUUACGUCAAU, GCACCUCGAUAUCAGCAUA,

AUUCUGAGGUCACGUUUAA, GGAGUGUGUUUGCGUGUCA

siGENOME Mouse Itgam (CD11b) (M-046775-01-0005, 16409) siRNA - SMARTpool:

GCACCUCGGUAUCAGCAUA, CCAGAGCACUGUCACUUAU,

CAAGGGCAACCUAUCAUUU, AAGAAGCCUUGGUUUGUAA

**Table S1.** Sequences of the primers used for RT-PCR

| Gene | sequence F primer 5' - 3' | sequence R primer 5' - 3' |
|---|---|---|
| HPRT | GCAGTACAGCCCCAAAATGG | AACAAAGTCTGGCCTGTATCCAA |
| TNF-α | TCTCATCAGTTCTATGGCCC | GGGAGTAGACAAGGTACAAC |
| IL-6 | GTTCTCTGGGAAATCGTGGA | TGTACTCCAGGTAGCTATGG |
| IL-1β | TTGACGGACCCCAAAAGATG | AGAAGGTGCTCATGTCCTCA |
| IL-10 | ATGCAGGACTTTAAGGGTTACTTG | TAGACACCTTGGTCTTGGAGCTTA |

**Supplementary Figures and Legends**

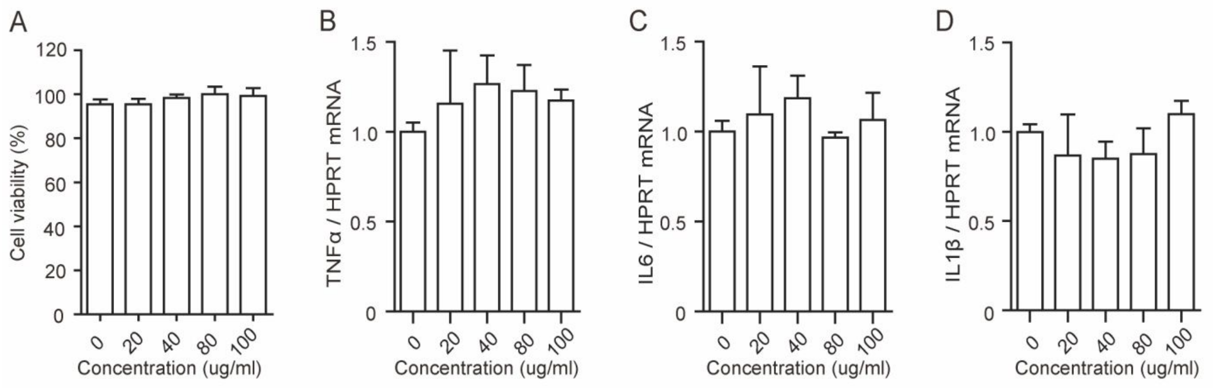


**Figure S1.** (A) Cell viability of BV2 cells treated with LPNPs at different concentrations for 24 h, the proportion of viable cells was evaluated using the CCK-8 assay according to the manufacturer's instructions (HY-K0301; Sigma-Aldrich) (n=5).(B-D) Pro-inflammatory cytokine gene expression in BV2 cells treated with different LPNPs concentrations for 24 h. (B) TNF-α, (C) IL-6 and (D) IL1β. n = 3. Data are presented as means ± SD and analyzed using one-way ANOVA.

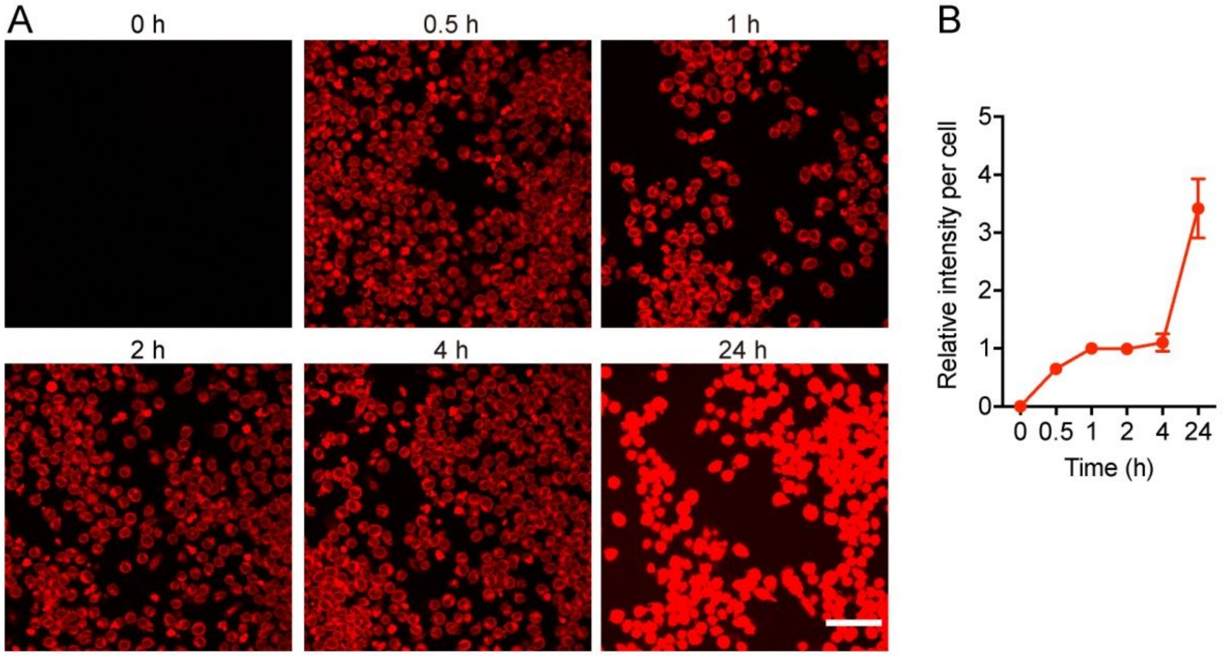


**Figure S2.** Accumulation of LPNP-siRNA in BV2 cells upon different duration of treatment *in vitro*. (A) LPNP-RhoB (red) accumulation in cultured microglial cells after incubation for 0.5 h, 1 h, 2 h, 4 h or 24 h. (B) Quantification of the relative fluorescent intensity of RhoB in microglial cells. These data are presented as means ± SD. Scale bar: 50 μm.

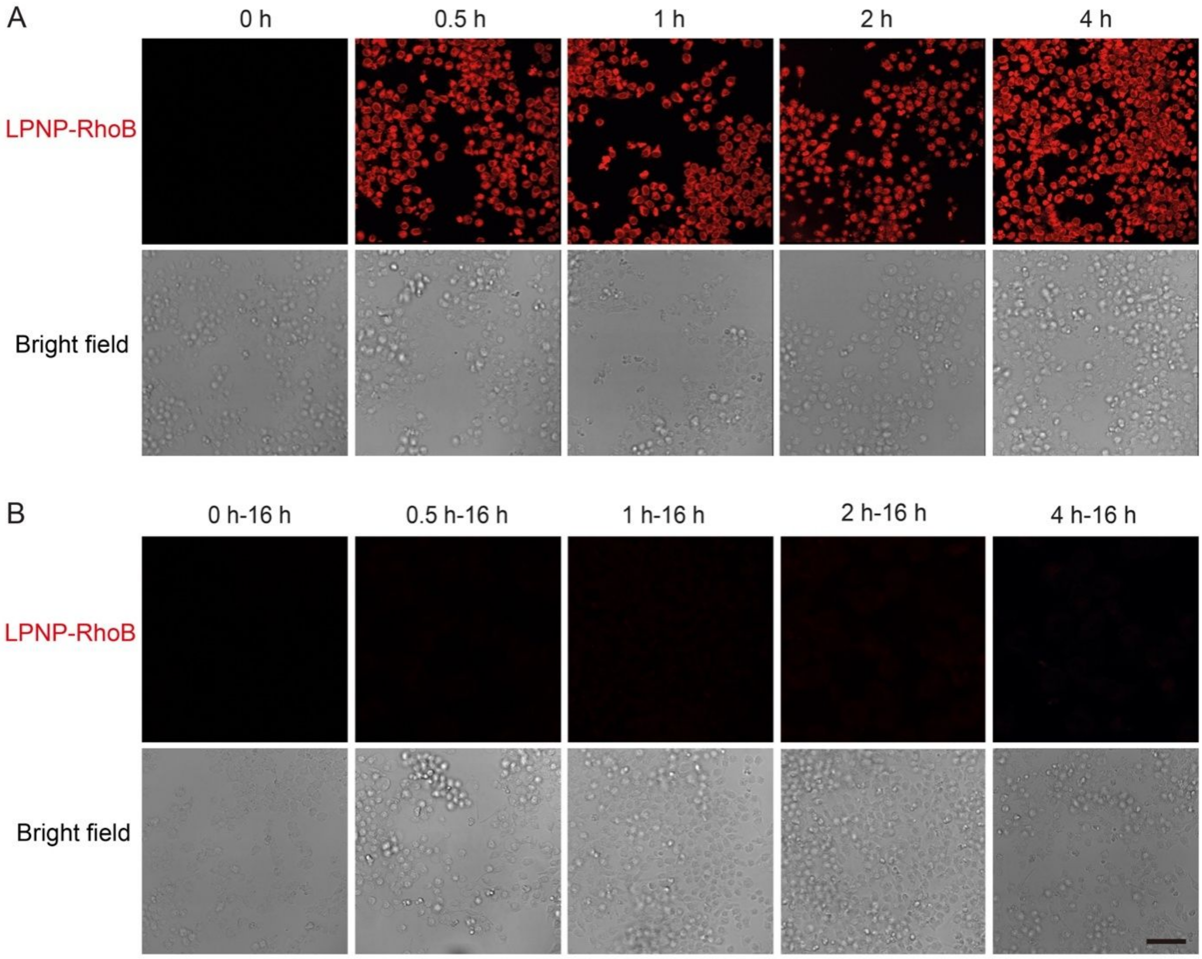


**Figure S3.** Accumulation and degradation of LPNP-RhoB in BV2 cells. (A) Representative images of BV2 cells treated with LPNP-RhoB-containing media for 0.5 h, 1 h, 2 h, or 4 h, respectively. (B) Representative images of BV2 cells when the LPNP-RhoB containing media were replaced with fresh media for another 16 h. Scale bars: 50 μm.

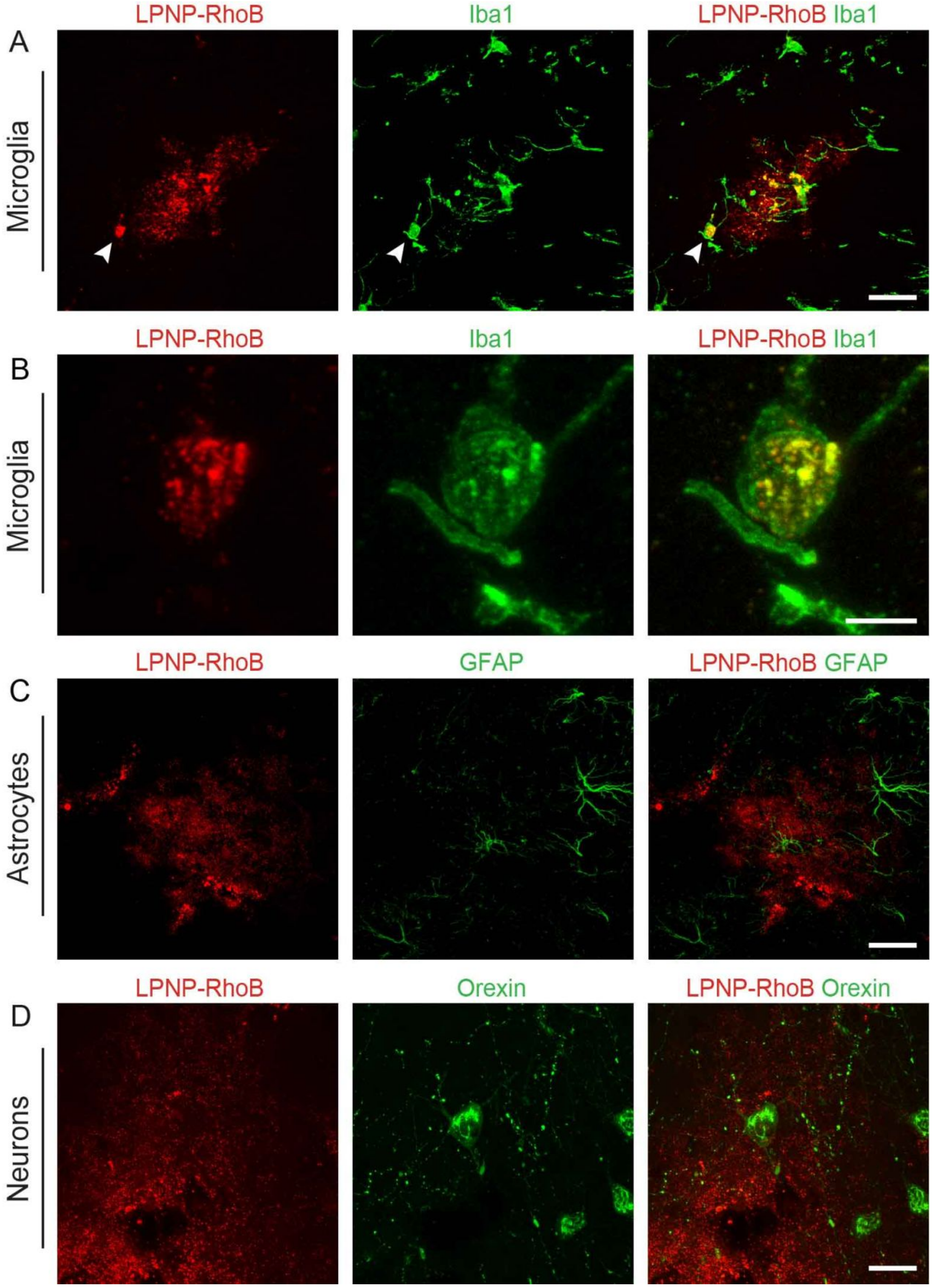


**Figure S4.** Specific accumulation of LPNP-RhoB in microglial cells in the rat hypothalamus. (A) Colocalization of LPNP-RhoB (red) and ionized calcium-binding adaptor molecule 1 immunoreactivity (Iba1-ir, green) in microglial cells. A higher magnificaitn of the microglia pointed by the white arrow head is shown in B. (C) No LPNP-RhoB (red) was detected inglial fibrillary acidic protein-ir(GFAP-ir, green) astrocytes. (D) No LPNP-RhoB (red) was detected in orexin-ir (green) neurons. n = 3. Scale bar: 20 μm in A, C and D, 5 μm in B.

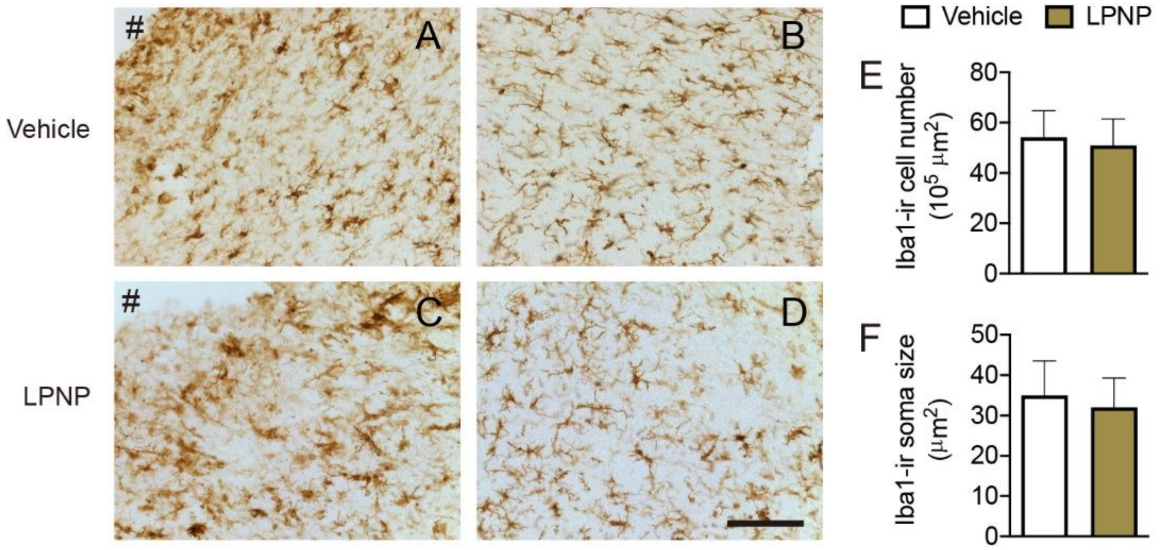


**Figure S5**. The response of microglial cells following LPNP injeciton in brain. (A - D) Representative images show Iba1-ir microglial cells near the injection spot (asterisk in A and C) and next to the injection spot (B and D) at the morphological level in the rat hypothalamus treated with LPNP for 20 h before sacrifice. (E, F) Quantification of the Iba1-ir microglial cell number and soma size (n = 4). The data are presented as means ± SD and analyzed using Student's *t*-test. Scale bar: 100 μm.

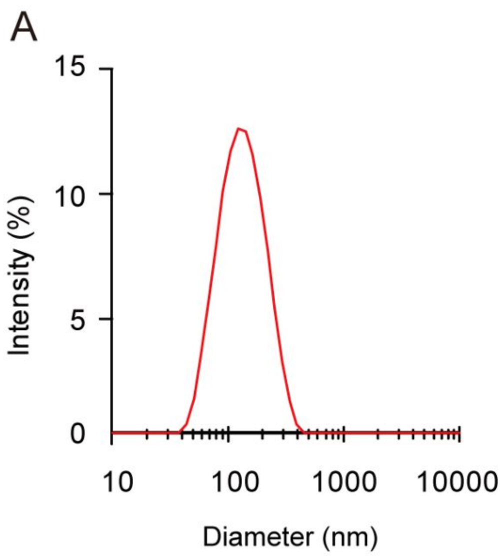


**Figure S6.** Diameter and zeta potential of LPNP-Au-555 as determined by dynamic light scattering. The average size of the LPNP-Au-555 is 113.6 nm (A), and the average surface charge of the LPNP-Au-555 is -19.7 mV (B).

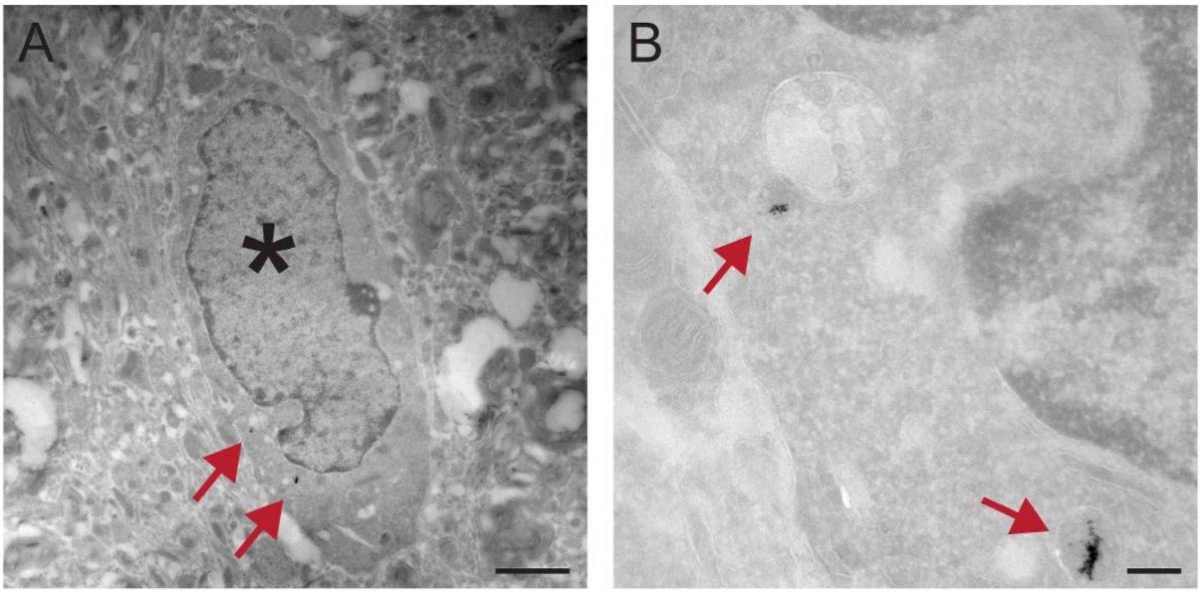


**Figure S7.** Characterization of the LPNP-Au nanoparticles accumulation in phagolysosomes by electron microscopy. (A) Arrows point to two phagolysosomes-like structures containing Au particles, these structures locate in a cytosolic area of a cell that does not possess a typical microglial dense and highly heterochromatin nucleus (asterisk shows the cell nucleus). (B) Higher magnification images of these phagolysosomes-like structures. Scale bar: 2 μm in A, 200 nm in B.

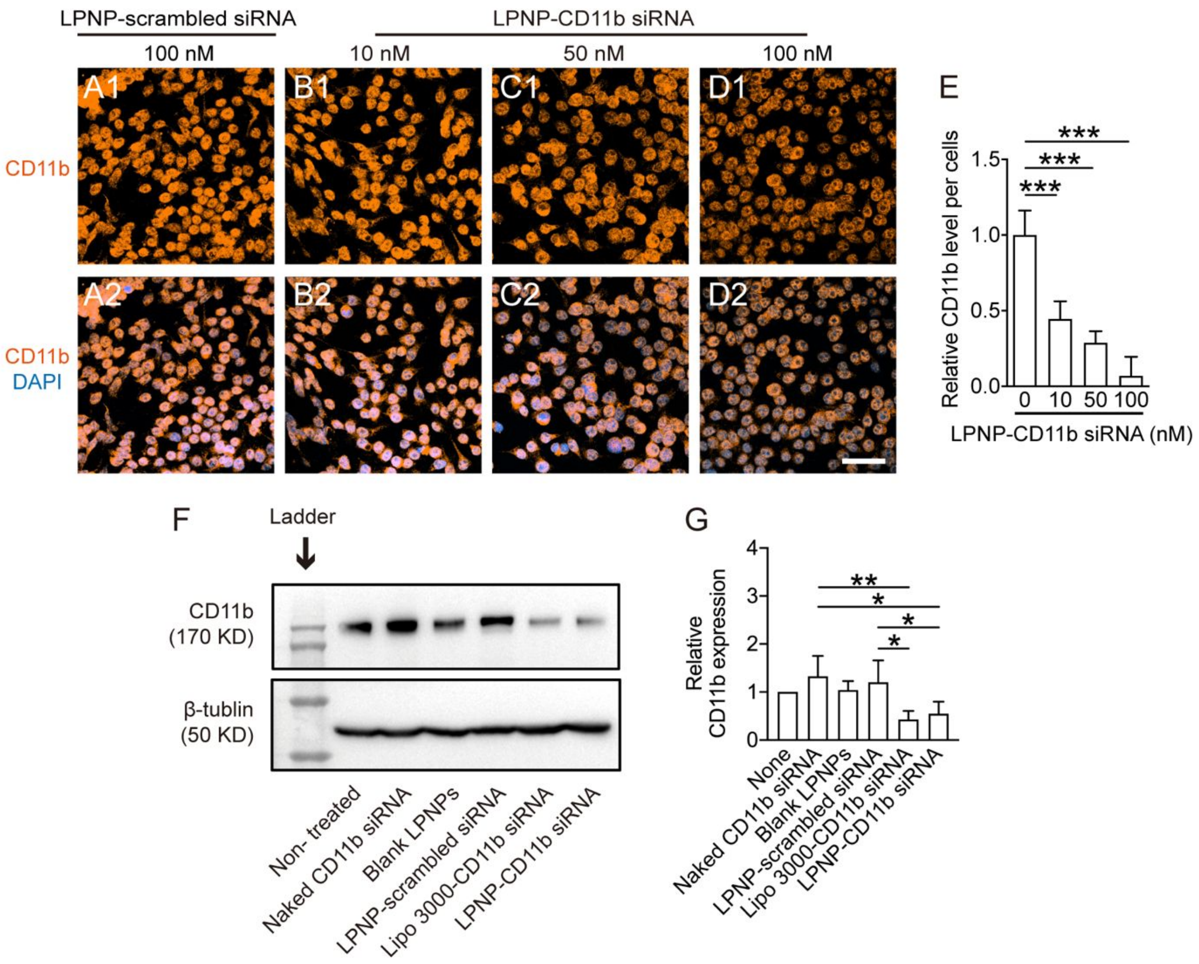


**Figure S8.** Evaluation of the silencing efficiency of LPNP-CD11b siRNA *in vitro*. (A-E) After the BV2 cells were treated with LPNP-CD11b siRNA at various siRNA concentrations (0, 10, 50 and 100 nM, with 0 nM representing LPNP-scrambled siRNA(100 nM, n=3), the expression of CD11b was assessed by immunocytochemical staining (A-D), the relative intensity CD11b immunofluorescence was quantified accordingly (E). (F) After the BV2 cells were treated with naked CD11b siRNA (100 nM), blank LPNPs (0.014 mg/ml), LPNP-scrambled siRNA (LPNP, 0.014 mg/ml; scrambled siRNA, 100 nM), Lipofectamine™ 3000-delivering CD11b siRNA (100 nM CD11b siRNA), and LPNP-CD11b siRNA (LPNP, 0.014 mg/ml; 100 nM CD11b siRNA) (n = 4), the expression of CD11b was assessed by western blotting. (G) The relative protein expression of CD11b was quantified accordingly. Data are presented as means ± SD and analyzed using one-way ANOVA in E and G. *$p < 0.05$, **$p < 0.01$, ***$p < 0.001$. Scale bar: 50 µm.
